# Supplementary material for: Novelty is not surprise: Human exploratory and adaptive behavior in sequential decision-making
Source: PLoS Comput Biol. 2021 Jun 3;17(6):e1009070. doi: 10.1371/journal.pcbi.1009070 (PMC8205159; doi:10.1371/journal.pcbi.1009070)
Supplement: S1 Text — (PDF) [file pcbi.1009070.s001.pdf]

Supplementary Information S1 Text: SurNoR and alternative algorithms

He A. Xu, Alireza Modirshanechi*, Marco P. Lehmann, Wulfram Gerstner, Michael H. Herzog

* alireza.modirshanechi@epfl.ch

Surprise-Novelty-Reward (SurNoR) algorithm

The SurNoR algorithm (Alg. A) combines surprise signals with novelty and reward so as to explore and learn the environment and exploit rewards. A simple block diagram of the algorithm is shown in Fig 4C of the main text. In the SurNoR algorithm, a model-based and a model-free branch interact with each other. The output of each branch is a pair of Q-values for estimated novelty and estimated reward. The model-based branch updates model-based Q-values using a world-model that is estimated online, while the model-free branch uses a surprise-modulated TD-learner for updating the model-free Q-values. Finally, actions are selected following a hybrid policy that combines model-free and model-based Q-values - see [1, 2] for similar approaches. In this section, we describe the SurNoR algorithm in detail. For the sake of clarity and coherence, we repeat here some details already explained in the main text.

Formalization of the environment. The state and the action at time t are random variables S_t and A_t which take values in the finite sets $\mathcal{S}$ and $\mathcal{A}$, respectively. In the particular case of our experiment, we have $\mathcal{S} = \{1, ..., 10, G\}$ and $\mathcal{A} = \{1, ..., 4\}$. Taking a Bayesian perspective, we consider the transition probability matrix as another random variable Θ, i.e.

$$\mathbb{P}(S_{t+1} = s' | S_t = s, A_t = a, \Theta = \theta) = \theta_{s,a}(s'). \tag{1}$$

where the values of $\theta_{s,a}(s')$ for combinations of s, a, and s' are unknown and needed to be estimated from experience. Since our environment is deterministic, except for the switch of two states before the start of block 2, the real transition probabilities are

$$\theta^{\text{real}}_{s,a}(s') = \delta(s', T(s, a)), \tag{2}$$

where $T(s, a)$ denotes the target state of the transition from state s given action a, and the Kronecker δ is defined as $\delta(x, x') = 1$ if $x = x'$ and zero otherwise; $T(s, a)$ corresponds to the arrows in Fig 1B and Fig 1D in the main text. The target state depends on the block number in our experiment. Note that $T(s, a)$ is unknown to the participants and to the SurNoR algorithm.

Definition of novelty. While a participant moves in the environment, the count

$$C^{(t)}_s = |\{t' : 1 \leq t' \leq t \text{ and } s_{t'} = s\}|$$

indicates how often state s has been encountered up to time t. We assume that at each time t, participants are able to estimate the empirical frequency $p_N^{(t)}(s)$ of encountering state $s \in \mathcal{S}$, formally defined as

$$p_N^{(t)}(s) = \frac{C_s^{(t)} + 1}{t + |\mathcal{S}|}, \tag{3}$$

where $|\mathcal{S}|$ is the total number of states (i.e., 11 for our experiment). Note that the participants know the total number of states, due to the pre-experiment introduction. The empirical frequency in Eq 3 is equal to the expected probability of observing state s given $s_{1:t}$ under the assumption of a uniform prior over the probabilities of observing different states: before the start of the experiment all $|\mathcal{S}|$ states have the same prior probability $p_N^{(0)}(s) = 1/|\mathcal{S}|$.

We define the novelty of the state s at time t as the negative logarithm of the empirical frequency

$$N^{(t)}(s) = -\log(p_N^{(t)}(s)). \tag{4}$$

In our algorithm, novelty acts just like an internally generated reward or exploration bonus (see subsection 'Formalizing model-based Q-values'). The main difference between our definition of novelty and most of the previously proposed measures of 'exploration bonus' [3–9] is in their dependency upon states *and* actions: while the usual exploration bonus measures are functions of state-action pairs, we define our novelty as a function of states only. Our choice is more consistent with the behavior of participants in our experiment, since a reasonable strategy of participants is to visit the states that they rarely encounter in the experiment as opposed to testing all actions in all states. From this perspective, our novelty measure is similar to the exploration bonus proposed by Bellemare, et al. (2016) [10].

In three of our alternative algorithms (see Section 'Alternative algorithms') we use a state-of-the-art exploration strategy [8, 9] which defines exploration bonus (or internal reward) as a function of the pairs of states and actions. We compare these algorithms with SurNoR (see Fig 5 in the main text).

Model-based branch of SurNoR

The pseudocode for the model-based branch is shown in Alg. B. In this subsection, the details are explained.

World-model. The participants knew that there were 11 states and 4 possible actions in each state. However, they were not aware of the actual transition probability matrix. In particular, they did not know whether the environment is deterministic or stochastic. Therefore, we define a participant's model of the world as an approximation q of the posterior distribution of the transition probability matrix, similar to the approach of [11–13],

$$q^{(t)}(\theta) \approx \mathbb{P}(\Theta = \theta | S_{1:t} = s_{1:t}, A_{1:t-1} = a_{1:t-1}). \tag{5}$$

In the following, we call q the belief of the participant. We assume that a participant estimates the transition probabilities by a weighted average

$$\hat{\theta}^{(t)} = \mathbb{E}_{q^{(t)}}[\Theta] = \int \theta q^{(t)}(\theta) d\theta, \tag{6}$$

where the weighting factor is given by the belief $q^{(t)}$. For convenience, the transition probability $\hat{\theta}_{s,a}^{(t)}(s')$ is written as $p^{(t)}(s'|s,a)$ in the main text, e.g., in Eq 4 and Eq 3.

For exact Bayesian inference one needs to explicitly specify the generative model which governs the transition. Particularly, the dynamics of Θ over time should be known, e.g., whether it is fixed, continuously drifting, or experiencing abrupt changes [14–17]. However, rather than making explicit assumptions about the generative model as a starting point for exact Bayesian inference, we work with a general (parametric, see the next part) distribution $q^{(t)}$ which is updated by an appropriate learning algorithm after each observation, similar to approaches in machine learning [18, 19].

Beliefs as Dirichlet distributions. We assume that the transition probabilities from different state-action pairs are independent of each other, i.e.

$$q^{(t)}(\theta) = \prod_{s\in\mathcal{S},a\in\mathcal{A}} q^{(t)}(\theta_{s,a}), \tag{7}$$

where $\theta_{s,a}$ is defined as in Eq 1. As a natural[1] choice for a probability distribution over transition probabilities, we consider the belief $q^{(t)}(\theta_{s,a})$ to be a Dirichlet distribution with parameter $\alpha^{(t)}_{s,a}$:

$$q^{(t)}(\theta_{s,a}) = \mathrm{Dir}(\theta_{s,a}; \alpha^{(t)}_{s,a}). \tag{8}$$

As a result, at each time t, the belief of participants about their environment can be summarized in the set $\alpha^{(t)} = \{\alpha^{(t)}_{s,a}, \forall(s,a) \in \mathcal{S}\times\mathcal{A}\}$. We consider the parameter of the prior belief $q^{(1)}$ (i.e., $\alpha^{(1)}$) to be the same for all transitions, i.e.,

$$\alpha^{(1)} = \{\alpha^{(1)}_{s,a}(s') = \epsilon, \quad \forall(s,s',a) \in \mathcal{S}\times\mathcal{S}\times\mathcal{A}\}, \tag{9}$$

where $\epsilon > 0$ is a free parameter. With this choice of prior, $\hat{\theta}^{(1)}_{s,a}$ (i.e., the prior estimate of the transition probabilities from the pair of state s and action a) is a uniform distribution over states. Furthermore, the free parameter ϵ expresses how deterministic the transitions are from the point of view of a participant, i.e., smaller values of ϵ indicate a more deterministic interpretation of the environment.

Using a Dirichlet distribution for the belief $q^{(t)}$ and Eq 6, a participant's estimation of the transition probabilities is

$$\hat{\theta}^{(t)}_{s,a}(s') = \frac{\alpha^{(t)}_{s,a}(s')}{\sum_{\tilde{s}'\in\mathcal{S}} \alpha^{(t)}_{s,a}(\tilde{s}')}. \tag{10}$$

Note that, the pseudo-counts $\tilde{C}^{(t)}_{s,a\to s'}$ in Eq 3 of the main text is equal to $\alpha^{(t)}_{s,a}(s') - \epsilon$.

Definitions of surprise. We work with the 'Bayes Factor' surprise $\mathbf{S}_{\mathrm{BF}}$ [14]. Consider the transition initiated at time t, i.e., $(S_t = s, A_t = a) \to (S_{t+1} = s')$. The Bayes Factor surprise corresponding to this transition is [14]

$$\mathbf{S}^{(t+1)}_{\mathrm{BF}} = \frac{\hat{\theta}^{(1)}_{s,a}(s')}{\hat{\theta}^{(t)}_{s,a}(s')}. \tag{11}$$

Due to the particular form of the prior $q^{(1)}$ that we chose, $\hat{\theta}^{(1)}_{s,a}(s')$ is constant. As a result, the surprise $\mathbf{S}^{(t+1)}_{\mathrm{BF}}$ at time $t+1$ is proportional to the inverse of the estimated probability $\hat{\theta}^{(t)}_{s,a}(s')$ of the transition initiated at time t. In Eq 5 of the main text, $\hat{\theta}^{(t)}_{s,a}(s')$ is written as $p^{(t)}(s'|s,a)$, and $\hat{\theta}^{(1)}_{s,a}(s')$ is written as $p_{\mathrm{reset}}(s'|s,a)$.

[1] If transition probabilities are stationary and have a uniform (or in general any Dirichlet) prior, exact Bayesian inference yields a Dirichlet distribution.

We note that in the particular case of our behavioral paradigm, the Shannon surprise [20] is just the shifted logarithm of the 'Bayes Factor' surprise, i.e., $\mathbf{S}_{\mathrm{Sh}}^{(t+1)} = \log \mathbf{S}_{\mathrm{BF}}^{(t+1)} + \log |\mathcal{S}|$. Furthermore, the state prediction error (SPE) [1] is an increasing function of the 'Bayes Factor' surprise, i.e., $\mathrm{SPE}^{(t+1)} = 1 - \frac{1}{|\mathcal{S}|\, \mathbf{S}_{\mathrm{BF}}^{(t+1)}}$. Hence, surprise-modulated learning rates in the SurNoR algorithm can alternatively be expressed in terms of $\mathbf{S}_{\mathrm{BF}}^{(t+1)}$ or $\mathbf{S}_{\mathrm{Sh}}^{(t+1)}$ or $\mathrm{SPE}^{(t+1)}$.

Surprise-modulated update of the belief. Learning the world-model corresponds to updating the parameters of the Dirichlet distribution after each transition. Consider the transition $(S_t = s, A_t = a) \to (S_{t+1} = s')$ initiated at time t which generates a surprise $\mathbf{S}_{\mathrm{BF}}^{(t+1)}$ at time $t+1$. The surprise-modulated adaptation rate is defined as [14]

$$\gamma(\mathbf{S}_{\mathrm{BF}}^{(t+1)}, m) = \frac{m\mathbf{S}_{\mathrm{BF}}^{(t+1)}}{1 + m\mathbf{S}_{\mathrm{BF}}^{(t+1)}} \in [0, 1], \tag{12}$$

where $m > 0$ is a positive free parameter. The parameter m controls the sharpness of the transition.

With this modulated adaptation rate, the change in a participant's belief is given by an update of the Dirichlet parameters $\alpha_{\tilde{s},\tilde{a}}^{(t+1)}(\tilde{s}')$ for all $(\tilde{s}, \tilde{s}', \tilde{a}) \in \mathcal{S} \times \mathcal{S} \times \mathcal{A}$ [14]

$$\alpha_{\tilde{s},\tilde{a}}^{(t+1)}(\tilde{s}') = \begin{cases} (1 - \gamma_{t+1})\alpha_{\tilde{s},\tilde{a}}^{(t)}(\tilde{s}') + \gamma_{t+1}\alpha^{(1)}(\tilde{s}') + \delta(s', \tilde{s}') & \text{if} \quad \tilde{s} = s, \tilde{a} = a \\ \alpha_{\tilde{s},\tilde{a}}^{(t)}(\tilde{s}') & \text{otherwise} \end{cases}, \tag{13}$$

where $\gamma_{t+1} = \gamma(\mathbf{S}_{\mathrm{BF}}^{(t+1)}, m)$. The update rule becomes the same as the one in Eq 6 of the main text if we replace $\alpha_{\tilde{s},\tilde{a}}^{(t)}(\tilde{s}')$ by $\tilde{C}_{\tilde{s},\tilde{a}\to\tilde{s}'}^{(t)} + \epsilon$. The update rule expresses the new belief as a mix between two possibilities, represented by the current parameters $\alpha_{\tilde{s},\tilde{a}}^{(t)}(\tilde{s}')$ and the prior $\alpha^{(1)}(\tilde{s}')$, weighted with $1 - \gamma_{t+1}$ and γ_{t+1}, respectively. In the case of a large surprise, the value of γ_{t+1} is close to one, and as a result, the current parameters are forgotten. The update makes a step based on the currently observed transition, expressed by the Kronecker-δ in the first line. The parameters of transitions from the pairs of the states and actions different form the current one (i.e., s and a) are not changed (second line). The update rule of Eq 13 is called Variational Surprise Minimizing Learning (VarSMiLe) rule in [14].

Formalizing model-based Q-values. The world-model of the participants is summarized by their beliefs $q^{(t)}(\theta)$ about the transition matrix of the environment. The belief is used for evaluation of two sets of Q-values [21], one for novelty N and the other one for the external reward R.

Novelty $N^{(t)}(s)$ of state s at time t (cf. Eq 4) is useful to guide behavior during exploration. Analogous to the common framework in reinforcement learning [21] where information of a reward at state s' is propagated by the Bellman equation to states $s \neq s'$, we use a Bellman equation to propagate the novelty of state s' to other states $s \neq s'$ by using the model of the world. More specifically, for the model-based branch, we assign to each state-action pair a novelty-based value $Q_{\mathrm{MB,N}}^{(t)}(s, a)$ which is an estimation of the accumulated future discounted novelty that can be gained by taking action a in state s. The Bellman equation is

$$Q_{\mathrm{MB,N}}^{(t)}(s, a) = \sum_{s' \in \mathcal{S}} \hat{\theta}_{s,a}^{(t)}(s') \Big(N^{(t)}(s') + \lambda_N \max_{a' \in \mathcal{A}} Q_{\mathrm{MB,N}}^{(t)}(s', a') \Big), \tag{14}$$

where $\hat{\theta}_{s,a}^{(t)}(s')$ are the estimated transition probabilities, and $\lambda_N \in [0, 1]$ is a discount factor for novelty. The Bellman equation assigns a value to the action a in state s as

long as a novel state is likely to be reached within the next few steps - even if the immediately neighboring states are not novel. The discount rate λ_N controls the time horizon of 'future novelty'. For $\lambda_N \to 0$, only the novelty of the immediately following state matters; for $\lambda_N \to 1$, the time horizon becomes infinitely long.

Rewards $R(s)$ of states $s \in \mathcal{S}$ guide behavior during exploitation. In the theory of reinforcement learning, reward information is summarized in values $Q^{(t)}_{\text{MB,R}}(s,a)$ that are estimations of the accumulated future discounted reward that can be collected when starting at state s with action a. The Q-values are given by the Bellman equation

$$Q^{(t)}_{\text{MB,R}}(s,a) = \sum_{s' \in \mathcal{S}} \hat{\theta}^{(t)}_{s,a}(s') \Big(R(s') + \lambda_R \max_{a' \in \mathcal{A}} Q^{(t)}_{\text{MB,R}}(s',a') \Big), \tag{15}$$

where $\lambda_R \in [0,1]$ is the discount factor for reward, which is not necessarily equal to the discount factor for novelty λ_N. Note that in our environment $R(s) = 0$ at all states except at the goal. Since the scale of the reward is arbitrary, we set $R(s_{Goal}) = 1$. As a result, the reward function is $R(s) = \delta(s, s_{Goal})$.

The total model-based Q-value is a linear combination of the Q-values for novelty $Q^{(t)}_{\text{MB,N}}(s,a)$ and reward $Q^{(t)}_{\text{MB,N}}(s,a)$,

$$Q^{(t)}_{\text{MB}}(s,a) = Q^{(t)}_{\text{MB,R}}(s,a) + \beta_N Q^{(t)}_{\text{MB,N}}(s,a), \tag{16}$$

where $\beta_N \geq 0$ is a free parameter controlling the trade-off between exploitation and exploration, i.e., between reward-seeking and novelty-seeking behavior.

In our model, β_N depends on whether participants are in the exploration phase or the exploitation phase. This dependency is simplified as follows: Since novelty is the main drive in the 1st episode of the 1st block, we keep β_N fixed at a value β_{N1} throughout this episode. However, at the end of the 1st episode of the 1st block, once participants have found the goal and do not need further exploration, we set $\beta_N = 0$ and keep it at zero for all remaining episodes of the 1st block.

Surprise increases rapidly at the first mismatch that participants face in the 1st episode of the 2nd block, when they encounter an unexpected transition. We hypothesize that the unexpected transitions make them realize that something has changed in the environment and they do not know anymore a path to the goal state and need to re-explore the environment and search for the goal in the absence of any external reward; hence, we assume that the huge surprise signal triggers renewed exploration and we therefore set $\beta_N = \beta_{N2}$ for the 1st episode of the 2nd block. With the same arguments as for the 1st block, we set β_N to zero for the remaining episodes of the 2nd block. β_{N1} and β_{N2} are free parameters of the model.

We also tested a variant of SurNoR with an additional free parameter $\beta_N = \beta_{N-2to5}$ for the weights of $Q^{(t+1)}_{\text{MF,N}}$ and $Q^{(t+1)}_{\text{MB,N}}$ in episodes 2-5 of blocks 1 and 2, but we did not find any significant improvement in the fit (difference in log-evidence = 15 ± 13).

Note that, for model comparison, we use the same assumptions for all other alternative algorithms that either seek novelty or uncertainty - see Section 'Alternative algorithms'.

Updating model-based Q-value. Since solving the non-linear equations 14 and 15 for computing two separate sets of model-based Q-values (i.e., $Q^{(t)}_{\text{MB,N}}(s,a)$ and $Q^{(t)}_{\text{MB,R}}(s,a)$ for all $(\tilde{s},\tilde{a}) \in \mathcal{S} \times \mathcal{A}$) is computationally costly, we use a variant (Algorithm D) of Prioritized Sweeping [21–23].

The idea of the algorithm, for example for updating $Q^{(t)}_{\text{MB,R}}(s,a)$, is to define a set of

$|\mathcal{S}|$ mirror variables $U_{\mathrm{R}}^{(t)}(s)$, and rewrite Eq 15 as

$$\begin{aligned} Q_{\mathrm{MB,R}}^{(t)}(s,a) &= \sum_{s' \in \mathcal{S}} \hat{\theta}_{s,a}^{(t)}(s')\Big(R(s') + \lambda_R U_{\mathrm{R}}^{(t)}(s')\Big) \\ U_{\mathrm{R}}^{(t)}(s') &= \max_{a' \in \mathcal{A}} Q_{\mathrm{MB,R}}^{(t)}(s',a'). \end{aligned} \tag{17}$$

At the transition from time step $t-1$ to time step t several iterations take place. The algorithm first puts $U_{\mathrm{R}}^{(t)}(s) = U_{\mathrm{R}}^{(t-1)}(s)$ and updates $Q_{\mathrm{MB,R}}^{(t)}(s,a)$ for all s, a with the current values of $U_{\mathrm{R}}^{(t)}(s)$ using the 1st equation. The size of the update step for the value of a state s' is measured as $\Delta V(s') = |U_{\mathrm{R}}^{(t)}(s') - \max_{a' \in \mathcal{A}} Q_{\mathrm{MB,R}}^{(t)}(s',a')|$. The states s' are then ordered in a priority queue with the state of biggest update step at the top. The algorithm updates the values of $U_{\mathrm{R}}^{(t)}(s')$ of the top priority state using the 2nd equation. This results in further updates $\Delta Q_{\mathrm{MB,R}}^{(t)}(s,a) = \hat{\theta}_{s,a}^{(t)}(s')\lambda_R \Delta V(s')$ for all s, a induced by the first equation. After these updates the priority list is resorted. Updating ends after T_{PS} iterations where $T_{\mathrm{PS}} \in \mathbb{N}$ is a free parameter of the algorithm.

The values of $Q_{\mathrm{MB,R}}^{(1)}(s,a)$, $U_{\mathrm{R}}^{(1)}(s)$, $Q_{\mathrm{MB,N}}^{(1)}(s,a)$, and $U_{\mathrm{N}}^{(1)}(s)$ are initialized consistent with the Bellman equations under the prior world-model (uniform distribution for all transitions) and the prior reward (zero) and novelty values ($\log|\mathcal{S}|$). For details, see Algorithms A and D.

SurNoR model-free branch

The pseudocode for the model-free branch is shown in Alg. C. In this subsection, the details are explained.

Formalizing model-free Q-values. Analogous to the model-based branch, we define $Q_{\mathrm{MF,R}}^{(t)}(s,a)$ and $Q_{\mathrm{MF,N}}^{(t)}(s,a)$ as values of the state-action pairs corresponding to the external reward R and novelty N, respectively. In contrast to the model-based branch, the model-free Q-values are updated using TD-learning [21, 24], for which the model of the world is not directly used - see the paragraph 'Updating model-free Q-values'.

Analogous to the total model-based Q-values, we define the total model-free Q-values as

$$Q_{\mathrm{MF}}^{(t)}(s,a) = Q_{\mathrm{MF,R}}^{(t)}(s,a) + \beta_N Q_{\mathrm{MF,N}}^{(t)}(s,a), \tag{18}$$

where $\beta_N \geq 0$ has the same value as the one used in Eq 16.

Reward and novelty prediction errors. A crucial signal in model-free reinforcement learning is the reward prediction error (RPE), defined as the difference between the expected 'reward' of a state-action pair and its real 'reward' [21]. Since we defined two separate sets of Q-values, one for the external reward and one for novelty (which plays the role of an 'internal reward'), we also define two separate corresponding prediction errors.

Consider the transition $(S_t = s, A_t = a) \to (S_{t+1} = s')$. The RPE at time $t+1$ is defined as

$$RPE_{t+1} = R(s') + \lambda_R \max_{a' \in \mathcal{A}} Q_{\mathrm{MF,R}}^{(t)}(s',a') - Q_{\mathrm{MF,R}}^{(t)}(s,a), \tag{19}$$

and similarly, the novelty prediction error (NPE) at time $t+1$ is defined as

$$NPE_{t+1} = N^{(t)}(s') + \lambda_N \max_{a' \in \mathcal{A}} Q_{\mathrm{MF,N}}^{(t)}(s',a') - Q_{\mathrm{MF,N}}^{(t)}(s,a), \tag{20}$$

where λ_R and λ_N are the same discount factors as the ones used in the model-based branch.

Eligibility trace. To keep track of the previously chosen state-action pairs, and to include them in the update rule, we use eligibility traces [21, 25, 26]. To have the most general setting, we define two separate eligibility traces, one for the external reward $e_R^{(t)}(s,a)$ and one for novelty (the internal reward) $e_N^{(t)}(s,a)$ for all state-action pairs (s,a). We initialize the eligibility traces at zero and reset their values to zero at the beginning of each episode. After the transition $(S_t = s, A_t = a) \rightarrow (S_{t+1} = s')$, the eligibility traces are updated to

$$\begin{aligned} e_R^{(t+1)}(s',a') &= \begin{cases} 1 & \text{if} \quad s' = s, a' = a \\ \lambda_R \mu_R e_R^{(t)}(s',a') & \text{otherwise} \end{cases} \\ e_N^{(t+1)}(s',a') &= \begin{cases} 1 & \text{if} \quad s' = s, a' = a \\ \lambda_N \mu_N e_N^{(t)}(s',a') & \text{otherwise}, \end{cases} \end{aligned} \tag{21}$$

where λ_R and λ_N are the discount factors defined above, and $\mu_N \in [0,1]$ and $\mu_R \in [0,1]$ are free parameters expressing how fast eligibility traces decay in time.

Surprise modulation of model-free learning rate. Usual TD learning algorithms use a constant (or decreasing in time) learning rate for updating Q-values [21]. However, the model-free branch of SurNoR has a learning rate modulated by the model-based branch. This novel interaction between model-based and model-free modules has not been explored by previous hybrid models in neuroscience, e.g., [1, 2].

We define the surprise modulated model-free learning rate ρ_t as

$$\rho_t = \rho_b + \gamma(\mathbf{S}_{\mathrm{BF}}^{(t)}, m)\delta\rho, \tag{22}$$

where $\gamma(\mathbf{S}_{\mathrm{BF}}^{(t)}, m)$ is the surprise modulated adaptation rate of the model-based branch defined in Eq 12, $\rho_b \in [0,1]$ is the baseline learning rate (when there is no surprise, i.e., if $\mathbf{S}_{\mathrm{BF}}^{(t)} = 0$), and $\delta\rho \in [0, 1-\rho_b]$ is the maximum possible variation of the learning rate due to the surprise modulation. As a result, the learning rate value ρ_t ranges between ρ_b (when $\mathbf{S}_{\mathrm{BF}}^{(t)} = 0$) and $\rho_b + \delta\rho$ (when $\mathbf{S}_{\mathrm{BF}}^{(t)} \rightarrow \infty$).

Updating model-free Q-value. The model-free Q-values for external reward are initialized to zero, $Q_{\mathrm{MF,R}}^{(1)}(s,a) = 0$ for all s, a. This initialization avoids any potential bias towards optimistic initialization (OI). The reason for this choice is to have novelty as the only exploration drive during the 1st episode of the 1st block. We separately test the effect of the initialization of reward-based Q-values in three alternative algorithm which use OI of $Q_{\mathrm{MF,R}}^{(1)}(s,a)$ as a drive for exploration [21] - see Section 'Alternative algorithms'. However, to consider the most general case, we initialize the model-free Q-values for novelty at $Q_{\mathrm{MF,N}}^{(1)}(s,a) = Q_{N0}$ with a free parameter $Q_{N0} \geq 0$.

At each time step $t+1$, the model-free Q-values are updated with a TD-learning algorithm

$$\begin{aligned} Q_{\mathrm{MF,R}}^{(t+1)}(s,a) &= Q_{\mathrm{MF,R}}^{(t)}(s,a) + \rho_{t+1} e_R^{(t+1)}(s,a) RPE_{t+1} \\ Q_{\mathrm{MF,N}}^{(t+1)}(s,a) &= Q_{\mathrm{MF,N}}^{(t)}(s,a) + \rho_{t+1} e_N^{(t+1)}(s,a) NPE_{t+1}. \end{aligned} \tag{23}$$

for all $(s,a) \in \mathcal{S} \times \mathcal{A}$.

Hybrid policy

The policy for action selection is based on a linear combination of Q-values, similar to [1, 2]. We use a softmax policy [21] and consider the probability of choosing action a in state s as

$$\pi(A_t = a | S_t = s) = \frac{1}{Z(s)} \exp\left\{\beta \left[\omega \left(\omega_{\mathrm{scale}} Q_{\mathrm{MF}}^{(t)}(s,a)\right) + (1-\omega) Q_{\mathrm{MB}}^{(t)}(s,a)\right]\right\}, \tag{24}$$

where $Z(s)$ is the normalization constant (that ensures that $\sum_a \pi(A_t = a|S_t = s) = 1$), $\omega_{\text{scale}} \geq 0$ is a free parameter to correct the potentially different scaling of the model-based and model-free values, and $\omega \in [0, 1]$ is a free parameter to balance the relative contribution of the model-based and model-free branches on the policy. When $\omega = 1$, the policy is purely model-free (but includes the effect of surprise modulation on the TD-learning learning rate), and when $\omega = 0$, the policy is purely model-based. Note that ω_{MF} and ω_{MB} mentioned in the main texts are equal to $\omega \times \omega_{\text{scale}}$ and $1 - \omega$, respectively. The reverse temperature $\beta \geq 0$ controls the sharpness of policy (the larger β the more deterministic is the policy).

As it was shown by [1], ω can vary in time. Specific to our experiment, we consider ω to be piece-wise constant in time: 1. $\omega = \omega_{11}$ for the 1st episode of the 1st block, when participants are in the pure exploration phase, 2. $\omega = \omega_{12}$ for the 1st episode of the 2nd block, when the goal is lost, and 3. $\omega = \omega_0$ for the rest of the experiments (i.e., episodes 2 to 5 for both blocks), when participants are in the exploitation phase. Moreover, we allow the value of β to be different for the 1st and the 2nd block, β_1 and β_2 respectively. By doing so, we allow the model to change its confidence in action selection after observing the sudden change in the environment.

Note that, for model comparison, we use the same assumptions for all other alternative algorithms that use hybrid policy - see Section 'Alternative algorithms'.

Summary of free parameters

SurNoR has 18 free parameters, summarized as

$$\{\epsilon, m, \lambda_R, \lambda_N, \beta_1, \beta_2, \beta_{N1}, \beta_{N2}, T_{\text{PS}}, \mu_R, \mu_N, Q_{N0}, \rho_b, \delta\rho, \omega_{\text{scale}}, \omega_0, \omega_{11}, \omega_{12}\}. \tag{25}$$

ϵ is used for initialization of the belief in Eq 9. m is used for modulation of the adaptation rate in Eq 12. λ_R and λ_N are discount factors used in the definitions and the updates of Q-values. β_1 and β_2 are the inverse temperatures controlling the sharpness of the hybrid policy in Eq 24. β_{N1} and β_{N2} are used for balancing novelty against external reward in equations 16 and 18. T_{PS} is used for Prioritized Sweeping in Algorithm D. μ_R and μ_N are used for controlling the decay of eligibility traces in Eq 21. Q_{N0} is used for initialization of $Q_{\text{MF,N}}$. ρ_b and $\delta\rho$ are used for the baseline learning rate of the model-free branch and its surprise modulation in Eq 22. ω_{scale} is used for correcting the potential different scaling of the model-based and model-free values in Eq 24, and ω_0, ω_{11}, and ω_{12} are used for balancing model-free against model-based in the hybrid policy of Eq 24.

Algorithm A Pseudocode for SurNoR

Specify $\mathcal{S}$ and $\mathcal{A}$
Specify Episode (Epi) and Block
\# Parameter specification
Specify $\{\epsilon, m, \lambda_R, \lambda_N, \beta_1, \beta_2, \beta_{N1}, \beta_{N2}, T_{\text{PS}}, \mu_R, \mu_N, Q_{N0}, \rho_b, \delta\rho, \omega_{\text{scale}}, \omega_0, \omega_{11}, \omega_{12}\}$.
if Block $= 1$ and Epi $= 1$, **then** Put $\beta = \beta_1$, $\omega = \omega_{11}$ and $\beta_N = \beta_{N1}$.
if Block $= 1$ and Epi $\neq 1$, **then** Put $\beta = \beta_1$, $\omega = \omega_0$ and $\beta_N = 0$.
if Block $= 2$ and Epi $= 1$, **then** Put $\beta = \beta_2$, $\omega = \omega_{12}$ and $\beta_N = \beta_{N2}$.
if Block $= 2$ and Epi $\neq 1$, **then** Put $\beta = \beta_2$, $\omega = \omega_0$ and $\beta_N = 0$.
\# Initialization
Put $e_R^{(1)}(s,a) = e_N^{(1)}(s,a) = 0$, $\forall (s,a) \in \mathcal{S} \times \mathcal{A}$.
if Epi $= 1$ and Block $= 1$ **then**
    Put $C_s^{(1)} = 0$, $U_R^{(1)}(s) = 0$, $U_N^{(1)}(s) = \frac{\log(|\mathcal{S}|)}{1-\lambda}$, $\forall s \in \mathcal{S}$.
    Put $Q_{\text{MB,R}}^{(1)}(s,a) = 0$, $Q_{\text{MB,N}}^{(1)}(s,a) = U_N^{(1)}(s)$, $\forall (s,a) \in \mathcal{S} \times \mathcal{A}$.
    Put $Q_{\text{MF,R}}^{(1)}(s,a) = 0$, $Q_{\text{MF,N}}^{(1)}(s,a) = Q_{N0}$, $\forall (s,a) \in \mathcal{S} \times \mathcal{A}$.
    Put $\alpha_{s,a}^{(1)}(s') = \epsilon$, $\forall (s,s',a) \in \mathcal{S} \times \mathcal{S} \times \mathcal{A}$.
else
    Initialize $C_s^{(1)}$, $U_R^{(1)}(s)$, $U_N^{(1)}(s)$, $Q_{\text{MB,R}}^{(1)}(s,a)$, $Q_{\text{MB,N}}^{(1)}(s,a)$, $Q_{\text{MF,R}}^{(1)}(s,a)$, $Q_{\text{MF,N}}^{(1)}(s,a)$ and $\alpha_{s,a}^{(1)}(s')$ with their latest values in the previous Episode.
Initialize state $S_1 = s_1$ and update counts $C_s^{(1)} \leftarrow C_s^{(1)} + \delta(s, s_1)$.
$t \leftarrow 1$.
\# Going through the task
while $s_t \neq s_{Goal}$ **do**
\# Making action
    Compute $Q_{\text{MF}}^{(t)}(s,a) = Q_{\text{MF,R}}^{(t)}(s,a) + \beta_N Q_{\text{MF,N}}^{(t)}(s,a)$.
    Compute $Q_{\text{MB}}^{(t)}(s,a) = Q_{\text{MB,R}}^{(t)}(s,a) + \beta_N Q_{\text{MB,N}}^{(t)}(s,a)$.
    Sample a_t from $\pi(A_t = a | S_t = s) \propto \exp\left\{\beta \left[\omega \left(\omega_{\text{scale}} Q_{\text{MF}}^{(t)}(s,a)\right) + (1-\omega)\, Q_{\text{MB}}^{(t)}(s,a)\right]\right\}$.
    Observe $S_{t+1} = s_{t+1}$.
\# Updating internal variables
    Update counts $C_s^{(t+1)} = C_s^{(t)} + \delta(s, s_{t+1})$ and novelty $N^{(t+1)}(s) = \log \frac{t+|\mathcal{S}|}{C_s^{(t+1)}+1}$.
    Update $\alpha^{(t+1)}$, $U_R^{(t+1)}$, $U_N^{(t+1)}$, $Q_{\text{MB,R}}^{(t+1)}$, and $Q_{\text{MB,N}}^{(t+1)}$ using the model-based branch in Alg. B.
    Update $e_N^{(t+1)}$, $e_R^{(t+1)}$, $Q_{\text{MF,R}}^{(t+1)}$, and $Q_{\text{MF,N}}^{(t+1)}$ using the model-free branch in Alg. C.
\# Going to the next step
    $t \leftarrow t + 1$.

Algorithm B Pseudocode for the model-based branch of SurNoR

Surprise and adaptation rate
Compute $\mathbf{S}^{(t+1)} = \hat{\theta}^{(1)}_{s_t,a_t}(s_{t+1}) / \hat{\theta}^{(t)}_{s_t,a_t}(s_{t+1})$.
Compute $\gamma_{t+1} = m\mathbf{S}^{(t+1)} / \big(1 + m\mathbf{S}^{(t+1)}\big)$.
Updating the belief
Update $\alpha^{(t+1)}_{s_t,a_t}(s) = (1-\gamma_{t+1})\alpha^{(t)}_{s_t,a_t}(s) + \gamma_{t+1}\epsilon + \delta(s_{t+1}, s)$, $\forall s \in \mathcal{S}$.
Update $\alpha^{(t+1)}_{s,a}(s') = \alpha^{(t)}_{s,a}(s')$, $\forall s \neq s_t, a \neq a_t$, and $s' \in \mathcal{S}$.
Update $\hat{\theta}^{(t+1)}$ as $\hat{\theta}^{(t+1)}_{s,a}(s') = \alpha^{(t+1)}_{s,a}(s') / \sum_{\tilde{s}' \in \mathcal{S}} \alpha^{(t+1)}_{s,a}(\tilde{s}')$.
Updating the values
Update $Q^{(t+1)}_{\mathrm{MB,N}}(s,a)$ and $U^{(t+1)}_{N}(s)$ using Alg. D and $N^{(t+1)}(s)$ as rewards.
if Epi $= 1$ and Block $= 1$ and $s_t \neq s_{Goal}$ **then**
    Update $Q^{(t+1)}_{\mathrm{MB,R}}(s,a) = U^{(t+1)}_{R}(s) = 0$.
else
    Update $Q^{(t+1)}_{\mathrm{MB,R}}(s,a)$ and $U^{(t+1)}_{R}(s)$ using Alg. D and $R(s) = \delta(s, s_{Goal})$ as rewards.

Algorithm C Pseudocode for the model-free branch of SurNoR

Surprise-modulated learning rate
Compute $\rho_{t+1} = \rho_b + \gamma_{t+1}\delta\rho$.
Prediction errors
Compute $RPE_{t+1} = R(s_{t+1}) + \lambda_R \max_{a' \in \mathcal{A}} Q^{(t)}_{\mathrm{MF,R}}(s_{t+1}, a') - Q^{(t)}_{\mathrm{MF,R}}(s_t, a_t)$.
Compute $NPE_{t+1} = N^{(t)}(s_{t+1}) + \lambda_N \max_{a' \in \mathcal{A}} Q^{(t)}_{\mathrm{MF,N}}(s_{t+1}, a') - Q^{(t)}_{\mathrm{MF,N}}(s_t, a_t)$.
Update of the eligibility traces
Update $e^{(t+1)}_N(s_t, a_t) = 1$, and $e^{(t+1)}_N(s,a) = \lambda_N \mu_N e^{(t)}_N(s,a)$, $\forall s \neq s_t, a \neq a_t$.
Update $e^{(t+1)}_R(s_t, a_t) = 1$, and $e^{(t+1)}_R(s,a) = \lambda_R \mu_R e^{(t)}_R(s,a)$, $\forall s \neq s_t, a \neq a_t$.
TD-learners
Update $Q^{(t+1)}_{\mathrm{MF,R}}(s,a) = Q^{(t)}_{\mathrm{MF,R}}(s,a) + \rho_{t+1} e^{(t+1)}_R(s,a) RPE_{t+1}$, $\forall s \in \mathcal{S}$ and $a \in \mathcal{A}$.
Update $Q^{(t+1)}_{\mathrm{MF,N}}(s,a) = Q^{(t)}_{\mathrm{MF,N}}(s,a) + \rho_{t+1} e^{(t+1)}_N(s,a) NPE_{t+1}$, $\forall s \in \mathcal{S}$ and $a \in \mathcal{A}$.

Algorithm D Pseudocode for the modified version of Prioritized Sweeping Algorithm for one time-step at time $t+1$

\# Specifying whether the update is for the internal or the external reward

Put $\lambda = \lambda_R$ for reward and $\lambda = \lambda_N$ for novelty.

Put $Q^{(t)} = Q^{(t)}_{\text{MB,R}}$, $U^{(t)} = U^{(t)}_R$, and Reward $= R$ for reward, and put $Q^{(t)} = Q^{(t)}_{\text{MB,N}}$, $U^{(t)} = U^{(t)}_N$, and Reward $= N^{(t+1)}$ for novelty.

\# Applying the effect of the latest observation on Q-values using previous U-values

for $(s,a) \in \mathcal{S} \times \mathcal{A}$ **do**

  $Q^{(t+1)}(s,a) = \sum_{s' \in \mathcal{S}} \hat{\theta}^{(t+1)}_{s,a}(s') \Big(\text{Reward}(s') + \lambda U^{(t)}(s') \Big)$

\# Making the priority queue

for $s \in \mathcal{S}$ **do**

  $U^{(t+1)}(s) = U^{(t)}(s)$

  $\text{Prior}(s) = |U^{(t+1)}(s) - \max_{a \in \mathcal{A}} Q^{(t+1)}(s,a)|$

\# Updating U-values for T_{PS} steps

for T_{PS} iterations **do**

  $s' = \arg\max_{s \in \mathcal{S}} \text{Prior}(s)$

  $\Delta V = \max_{a \in \mathcal{A}} Q^{(t+1)}(s',a) - U^{(t+1)}(s')$

  $U^{(t+1)}(s') = \max_{a \in \mathcal{A}} Q^{(t+1)}(s',a)$

  \# Applying the effect of the update of U-values on Q-values

  for $(s,a) \in \mathcal{S} \times \mathcal{A}$ **do**

    $Q^{(t+1)}(s,a) \leftarrow Q^{(t+1)}(s,a) + \lambda \hat{\theta}^{(t+1)}_{s,a}(s') \Delta V$

  \# Updating the priority queue

  for $s \in \mathcal{S}$ **do**

    $\text{Prior}(s) = |U^{(t+1)}(s) - \max_{a \in \mathcal{A}} Q^{(t+1)}(s,a)|$

Alternative algorithms

To statistically test the effect of surprise and novelty, we implemented 12 alternative algorithms explained in this section. Their key features are summarized in Table A. The modified versions of SurNoR which do not seek novelty but assign negative reward to the most frequent states are explained at the end.

Model-based alternatives. Four out of 12 algorithms are purely model-based. They all use world-model and prioritized sweeping to calculate model-based Q-values. However, they have different approaches for learning the world-model and different strategies for exploration.

(i) MB+S+N: This algorithm has both features of SurNoR in using surprise modulation for model-building and novelty-seeking for exploration, but it does not use a parallel TD-learner. MB+S+N is a reduced version of SurNoR with $\mu_R = \mu_N = Q_{N0} = \rho_b = \delta\rho = \omega_{\text{scale}} = \omega_0 = \omega_{11} = \omega_{12} = 0$, which is equivalent to the model-based branch of SurNoR. MB+S+N has 9 free parameters $\{\epsilon, m, \lambda_R, \lambda_N, T_{PS}, \beta_1, \beta_2, \beta_{N1}, \beta_{N2}\}$.

(ii) MB+N: This algorithm is a modified version of MB+S+N; it uses novelty-seeking for exploration, but it does not have surprise modulation for learning the world-model. MB+N uses leaky integration to update the belief parameters (analogous to Eq 13),

$$\alpha^{(t+1)}_{\tilde{s},\tilde{a}}(\tilde{s}') = \begin{cases} \kappa_{\text{Leak}}\alpha^{(t)}_{\tilde{s},\tilde{a}}(\tilde{s}') + \delta(s', \tilde{s}') & \text{if} \quad \tilde{s} = s, \tilde{a} = a \\ \alpha^{(t)}_{\tilde{s},\tilde{a}}(\tilde{s}') & \text{otherwise} \end{cases}, \tag{26}$$

where $\kappa_{\text{Leak}} \in [0, 1]$ is a constant free parameter. Such a learning rule has been used previously to model human behavior [27–30]. Overall, MB+N has 9 free parameters $\{\epsilon, \kappa_{\text{Leak}}, \lambda_R, \lambda_N, T_{PS}, \beta_1, \beta_2, \beta_{N1}, \beta_{N2}\}$. It cannot be considered as a special case of SurNoR, but it can be implemented in the framework of the SurNoR algorithm by using Eq 26 instead of Eq 13 for updating the belief and by putting $m = \mu_R = \mu_N = Q_{N0} = \rho_b = \delta\rho = \omega_{\text{scale}} = \omega_{11} = \omega_{12} = \omega_0 = 0$.

(iii) MB+S+U: This algorithm is similar to MB+S+N; it uses surprise modulation for learning the world-model, but it seeks uncertainty instead of novelty for exploration. Following the ideas from [8, 9], we define a set of uncertainty-based Q-values, analogous to the SurNoR's novelty-based Q-values (Eq 14), as

$$Q^{(t)}_{\text{MB,U}}(s, a) = \sum_{s' \in \mathcal{S}} \hat{\theta}^{(t)}_{s,a}(s')\Big(- \log \hat{\theta}^{(t)}_{s,a}(s') + \lambda_U \max_{a' \in \mathcal{A}} Q^{(t)}_{\text{MB,U}}(s', a')\Big), \tag{27}$$

where $- \log \hat{\theta}^{(t)}_{s,a}(s')$, sometimes called surprisal (equal to Shannon surprise) is considered as the intrinsic reward of the transition $(s, a) \to s'$. The model MB+S+U is implemented by modifying SurNoR in 3 steps: 1. Replacing Eq 14 by Eq 27 and using $Q^{(t)}_{\text{MB,U}}(s, a)$ instead of $Q^{(t)}_{\text{MB,N}}(s, a)$ in all equations. 2. Replacing λ_N by λ_U, β_{N1} by β_{U1}, and β_{N2} by β_{U2}. 3. Putting $\mu_R = \mu_N = Q_{N0} = \rho_b = \delta\rho = \omega_{\text{scale}} = \omega_0 = \omega_{11} = \omega_{12} = 0$. MB+S+U has 9 free parameters $\{\epsilon, m, \lambda_R, \lambda_U, T_{PS}, \beta_1, \beta_2, \beta_{U1}, \beta_{U2}\}$.

(iv) MB+S+OI: This algorithm removes the novelty-seeking block of MB+S+N and uses optimistic initialization for exploration, i.e., it updates reward-based Q-values also in the 1st episode of block 1 even before observing the goal states. MB+S+OI can be implemented by modifying SurNoR in 2 steps: 1. Removing the 'if' condition in the lines 7-10 of Alg. B and keeping only line 10. 2. Putting $\lambda_N = \beta_{N1} = \beta_{N2} = \mu_R = \mu_N = Q_{N0} = \rho_b = \delta\rho = \omega_{\text{scale}} = \omega_0 = \omega_{11} = \omega_{12} = 0$. This algorithm has 6 free parameters $\{\epsilon, m, \lambda_R, T_{PS}, \beta_1, \beta_2\}$.

Model-free alternatives. Four out of 12 algorithms are model-free. All of them use a TD-learner for learning model-free Q-values. However, the ones with surprise-modulation are also equipped with a world-model, but the world model is not used for computing a set of model-based Q-values, and the policy is not hybrid.

(v) MF+S+N: This algorithm is equivalent to the model-free branch of SurNoR, but it also uses the world-model in the model-based branch for surprise-computation. MF+S+N can be seen as a reduced version of SurNoR by putting $T_{PS} = 0$ and $\omega_{\text{scale}} = \omega_{11} = \omega_{12} = \omega_0 = 1$. It has 13 free parameters $\{\epsilon, m, \lambda_R, \lambda_N, \beta_1, \beta_2, \beta_{N1}, \beta_{N2}, \mu_R, \mu_N, Q_{N0}, \rho_b, \delta\rho\}$.

(vi) MF+N: This algorithm is a reduced version of SurNoR by putting $m = T_{PS} = \delta\rho = 0$ and $\omega_{\text{scale}} = \omega_{11} = \omega_{12} = \omega_0 = \epsilon = 1$, which is equivalent to the model-free branch of SurNoR without any surprise modulation. It can also be seen as a modified version of the famous $Q(\lambda)$ algorithm [21] (with $\lambda = \mu_R$ in our notation) but with novelty as an exploration bonus (instead of optimistic initialization). The model MF+N has overall 10 free parameters $\{\lambda_R, \lambda_N, \beta_1, \beta_2, \beta_{N1}, \beta_{N2}, \mu_R, \mu_N, Q_{N0}, \rho_b\}$.

(vii) MF+S+U: The relation between MF+S+U and MB+S+U is the same as the relation between MF+S+N and MB+S+N. All the features of MF+S+U (including the surprise modulation of the learning rate of the model-free) except for its exploration strategy are the same as the ones of MF+S+N. For exploration, MF+S+U seeks uncertainty instead of novelty. Similar to what we did for MB+S+U, we followed the ideas from [8,9] and defined the uncertainty-based Q-values as in Eq 27. Then, we define the Uncertainty Prediction Error (UPE), analogous to the SurNoR's NPE (Eq 20), as

$$UPE_{t+1} = -\log \hat{\theta}^{(t)}_{s,a}(s') + \lambda_U \max_{a' \in \mathcal{A}} Q^{(t)}_{\text{MF,U}}(s', a') - Q^{(t)}_{\text{MF,U}}(s, a), \tag{28}$$

and then we update the uncertainty-based model-free Q-values as

$$Q^{(t+1)}_{\text{MF,U}}(s, a) = Q^{(t)}_{\text{MF,U}}(s, a) + \rho_{t+1} e^{(t+1)}_U(s, a) UPE_{t+1}, \tag{29}$$

where $e^{(t+1)}_U(s, a)$ is the uncertainty eligibility trace with a decay factor μ_U. Then MF+S+U can be implemented by modifying SurNoR in three steps: 1. Replacing $Q^{(t)}_{\text{MF,N}}(s, a)$ by $Q^{(t)}_{\text{MF,U}}(s, a)$ in all equations. 2. Replacing λ_N by λ_U, β_{N1} by β_{U1}, β_{N2} by β_{U2}, and μ_N by μ_U. 3. Putting $T_{PS} = 0$ and $\omega_{\text{scale}} = \omega_{11} = \omega_{12} = \omega_0 = 1$. The model MF+S+U has 13 free parameters $\{\epsilon, m, \lambda_R, \lambda_U, \beta_1, \beta_2, \beta_{U1}, \beta_{U2}, \mu_R, \mu_U, Q_{U0}, \rho_b, \delta\rho\}$.

(iix) MF+OI: This algorithm is our simplest algorithm, and neither surprise nor novelty is used in it. MF+OI is equivalent to $Q(\lambda)$ [21], with $\lambda = \mu_R$ in our notation. It uses optimistic initialization for exploration by putting $Q^{(0)}_{MF,R} = Q_{R0}$, where Q_{R0} is a free parameter. It can be seen as a modified version of SurNoR by initializing $Q^{(0)}_{MF,R} = Q_{R0}$ and putting $m = \lambda_N = \beta_{N1} = \beta_{N2} = T_{PS} = \mu_N = Q_{N0} = \delta\rho = 0$ and $\omega_{\text{scale}} = \omega_{11} = \omega_{12} = \omega_0 = \epsilon = 1$. The model MF+OI has overall 6 free parameters $\{\lambda_R, Q_{R0}, \rho_b, \beta_1, \beta_2, \mu_R\}$.

Hybrid alternatives. Three out of 12 algorithms are hybrid, meaning they use both model-free and model-based Q-values for decision-making.

(ix) Hyb+N: This algorithm uses MB+N and MF+N in parallel and combines their Q-values (in the same fashion as in SurNoR) in a hybrid policy. It has overall 17 free parameters $\{\epsilon, \kappa_{\text{Leak}}, \lambda_R, \lambda_N, \beta_1, \beta_2, \beta_{N1}, \beta_{N2}, T_{PS}, \mu_R, \mu_N, Q_{N0}, \rho_b, \omega_{\text{scale}}, \omega_{11}, \omega_{12}, \omega_0\}$.

(x) Hyb+S+U: This algorithm uses MB+S+U and MF+S+U in parallel and combines their Q-values (in the same fashion as in SurNoR) in a hybrid policy. Hyb+S+U is as complex as SurNoR and has overall 18 free parameters $\{\epsilon, m, \lambda_R, \lambda_U, \beta_1, \beta_2, \beta_{U1}, \beta_{U2}, T_{PS}, \mu_R, \mu_U, Q_{U0}, \rho_b, \delta\rho, \omega_{\text{scale}}, \omega_{11}, \omega_{12}, \omega_0\}$.

(xi) Hyb+S+OI: This algorithm uses MF+OI (but with surprise modulation of the learning rate of the model-free branch) and MB+S+OI in parallel and combines their Q-values (in the same fashion as in SurNoR) in a hybrid policy. Hyb+S+OI has overall 14 free parameters $\{\epsilon, m, \lambda_R, \beta_1, \beta_2, T_{PS}, \mu_R, Q_{R0}, \rho_b, \delta\rho, \omega_{\text{scale}}, \omega_{11}, \omega_{12}, \omega_0\}$.

Table A. Summary of the key features of all models.

	Algorithm	World-model	Hybrid-policy	Novelty	Surprise	Param.
i	MB+S+N	✓	✗	✓	✓	9
ii	MB+N	✓	✗	✓	✗	9
iii	MB+S+U	✓	✗	✗	✓	9
iv	MB+S+OI	✓	✗	✗	✓	6
v	MF+S+N	✓	✗	✓	✓	13
vi	MF+N	✗	✗	✓	✗	10
vii	MF+S+U	✓	✗	✗	✓	13
iix	MF+OI	✗	✗	✗	✗	6
ix	Hyb+N	✓	✓	✓	✗	17
x	Hyb+S+U	✓	✓	✗	✓	18
xi	Hyb+S+OI	✓	✓	✗	✓	14
xii	RC	✗	✗	✗	✗	0
xiii	BinaryNovelty	✓	✓	(✓)	✓	19
xiv	**SurNoR**	✓	✓	✓	✓	18

Null model. (xii) RC (Random Choice): According to this algorithm, participants choose actions with uniform distribution, i.e., each action is selected with a probability equal to $\frac{1}{|\mathcal{A}|} = 0.25$. We used this model as a reference to quantify the effect of our novelty-seeking exploration in the 1st episode of the 1st block. This algorithm does not have any free parameter.

Control modifications of SurNoR. (xiii) Binary Novelty: The two control algorithms mentioned in the main text are exactly the same as SurNoR except for a change in the intrinsic motivation signal that drives exploration. While in the SurNoR algorithm the continuous-valued novelty signal defined in Eq 3 and Eq 4 serves as the intrinsic reward, in the control algorithms the intrinsic reward of state s at time t is binary: in the first control algorithm it is considered to be -1 if the count $C_s^{(t)} \geq C_{\text{thr}}$ and 0 otherwise, where C_{thr} is a new free parameter, i.e., the algorithm considers the states that are encountered more than C_{thr} times as bad states and assigns a constant negative reward to them. Similarly, in the 2nd control algorithm, the intrinsic reward of state s at time t is considered to be -1 if state s is among the n most frequently encountered states and 0 otherwise, where n is a new free parameter, i.e., the algorithm considers the n most frequently encountered states as bad states. Therefore, the pseudo-code of the control algorithms is the same as the pseudo-code of SurNoR in Alg. A but with 2 modifications: (i) $U_N^{(1)}(s)$ is initialized at a value 0. (ii) The definition of novelty is changed to

$$N^{(t)}(s) = \begin{cases} -1 & \text{if} \quad C_s^{(t)} \geq C_{\text{thr}} \\ 0 & \qquad \text{otherwise} \end{cases} \tag{30}$$

for the 1st algorithm and to

$$N^{(t)}(s) = \begin{cases} -1 & \text{if} \quad C_s^{(t)} \in n\,\text{highest counts} \\ 0 & \qquad\qquad \text{otherwise} \end{cases} \tag{31}$$

for the 2nd algorithm. Overall, both algorithm have 19 free parameters, i.e., 18 free parameters of SurNoR plus C_{thr} for the 1st and n for the 2nd control algorithm.

References

1. Gläscher J, Daw N, Dayan P, O'Doherty JP. States versus rewards: dissociable neural prediction error signals underlying model-based and model-free reinforcement learning. Neuron. 2010;66(4):585–595.

2. Daw ND, Gershman SJ, Seymour B, Dayan P, Dolan RJ. Model-based influences on humans' choices and striatal prediction errors. Neuron. 2011;69(6):1204–1215.

3. Kolter JZ, Ng AY. Near-Bayesian exploration in polynomial time. In: Proceedings of the 26th Annual International Conference on Machine Learning. ACM; 2009. p. 513–520.

4. Martin J, Narayanan SS, Everitt T, Hutter M. Count-based exploration in feature space for reinforcement learning. In: Proceedings of the 26th International Joint Conference on Artificial Intelligence. AAAI Press; 2017. p. 2471–2478.

5. Little DYJ, Sommer FT. Learning and exploration in action-perception loops. Frontiers in neural circuits. 2013;7:37.

6. Mobin SA, Arnemann JA, Sommer F. Information-based learning by agents in unbounded state spaces. In: Advances in Neural Information Processing Systems; 2014. p. 3023–3031.

7. Pathak D, Agrawal P, Efros AA, Darrell T. Curiosity-driven exploration by self-supervised prediction. In: Proceedings of the IEEE Conference on Computer Vision and Pattern Recognition Workshops; 2017. p. 16–17.

8. Achiam J, Sastry S. Surprise-based intrinsic motivation for deep reinforcement learning. arXiv preprint arXiv:170301732. 2017;.

9. Burda Y, Edwards H, Pathak D, Storkey A, Darrell T, Efros AA. Large-Scale Study of Curiosity-Driven Learning. In: International Conference on Learning Representations; 2018.

10. Bellemare M, Srinivasan S, Ostrovski G, Schaul T, Saxton D, Munos R. Unifying count-based exploration and intrinsic motivation. In: Advances in Neural Information Processing Systems; 2016. p. 1471–1479.

11. Friston K. The free-energy principle: a unified brain theory? Nature reviews neuroscience. 2010;11(2):127.

12. Friston K, FitzGerald T, Rigoli F, Schwartenbeck P, Pezzulo G. Active inference: a process theory. Neural computation. 2017;29(1):1–49.

13. Faraji M, Preuschoff K, Gerstner W. Balancing new against old information: the role of puzzlement surprise in learning. Neural computation. 2018;30(1):34–83.

14. Liakoni V, Modirshanechi A, Gerstner W, Brea J. Learning in Volatile Environments with the Bayes Factor Surprise. Neural Computation. 2021;33(2):1–72. doi:10.1162/neco_a_01352.

15. Mathys C, Daunizeau J, Friston KJ, Stephan KE. A Bayesian foundation for individual learning under uncertainty. Frontiers in human neuroscience. 2011;5:39.

16. Nassar MR, Wilson RC, Heasly B, Gold JI. An approximately Bayesian delta-rule model explains the dynamics of belief updating in a changing environment. Journal of Neuroscience. 2010;30(37):12366–12378.

17. Behrens TE, Woolrich MW, Walton ME, Rushworth MF. Learning the value of information in an uncertain world. Nature neuroscience. 2007;10(9):1214.

18. Ozkan E, Smidl V, Saha S, Lundquist C, Gustafsson F. Marginalized adaptive particle filtering for nonlinear models with unknown time-varying noise parameters. Automatica. 2013;49(6):1566–1575.

19. Masegosa A, Nielsen TD, Langseth H, Ramos-López D, Salmerón A, Madsen AL. Bayesian models of data streams with hierarchical power priors. In: Proceedings of the 34th International Conference on Machine Learning-Volume 70. JMLR. org; 2017. p. 2334–2343.

20. Shannon C. A mathematical theory of communication. Bell System Technical Journal 27: 379-423 and 623–656. 1948;20.

21. Sutton RS, Barto AG. Reinforcement learning: An introduction. MIT press; 2018.

22. Van Seijen H, Sutton RS. Efficient planning in MDPs by small backups. In: Proc. 30th Int. Conf. Mach. Learn.; 2013. p. 1–3.

23. Brea J. Is prioritized sweeping the better episodic control? arXiv preprint arXiv:171106677. 2017;.

24. Watkins CJ, Dayan P. Q-learning. Machine learning. 1992;8(3-4):279–292.

25. Lehmann MP, Xu HA, Liakoni V, Herzog MH, Gerstner W, Preuschoff K. One-shot learning and behavioral eligibility traces in sequential decision making. Elife. 2019;8:e47463.

26. Gerstner W, Lehmann M, Liakoni V, Corneil D, Brea J. Eligibility traces and plasticity on behavioral time scales: experimental support of neohebbian three-factor learning rules. Frontiers in neural circuits. 2018;12.

27. Yu AJ, Cohen JD. Sequential effects: superstition or rational behavior? In: Advances in neural information processing systems; 2009. p. 1873–1880.

28. Meyniel F, Maheu M, Dehaene S. Human inferences about sequences: A minimal transition probability model. PLoS computational biology. 2016;12(12):e1005260.

29. Modirshanechi A, Kiani MM, Aghajan H. Trial-by-trial surprise-decoding model for visual and auditory binary oddball tasks. NeuroImage. 2019;196:302–317.

30. Maheu M, Dehaene S, Meyniel F. Brain signatures of a multiscale process of sequence learning in humans. Elife. 2019;8:e41541.
