# Supplementary material for: Novelty is not surprise: Human exploratory and adaptive behavior in sequential decision-making
Source: PLoS Comput Biol. 2021 Jun 3;17(6):e1009070. doi: 10.1371/journal.pcbi.1009070 (PMC8205159; doi:10.1371/journal.pcbi.1009070)
Supplement: S2 Text — (PDF) [file pcbi.1009070.s002.pdf]

# Supplementary Information S2 Text: EEG preprocessing and control analyses

He A. Xu, Alireza Modirshanechi*, Marco P. Lehmann, Wulfram Gerstner, Michael H. Herzog

* alireza.modirshanechi@epfl.ch

## PCA over Reward and RPE

We show that PCA on the two normalized variables Reward and RPE yields $R_+$ and $R_-$.

**Lemma.** For two random variables $X_1$ and $X_2$ with zero mean (i.e., $\mathbb{E}(X_1) = \mathbb{E}(X_2) = 0$), unit variance (i.e., $\mathbb{E}(X_1^2) = \mathbb{E}(X_2^2) = 1$), and correlation $r = \mathbb{E}(X_1 X_2)$, the new variables $X_+ = (X_1 + X_2)/\sqrt{2}$ and $X_- = (X_1 - X_2)/\sqrt{2}$ are the projections on the two normalized principal components of the correlation matrix.

*Proof:* The 2x2 correlation matrix $C$ has diagonal elements $c_{11} = c_{22} = 1$ because of the normalization of each variable to unit variance and off-diagonal elements $c_{21} = c_{12} = r$ according to the assumption and symmetry of correlations. The normalized eigenvectors of the correlation matrix are then $e_+ = (1,1)^T/\sqrt{2}$ and $e_- = (1,-1)^T/\sqrt{2}$ with eigenvalues $\lambda_\pm = 1 \pm r$. ■

Therefore, if Reward and RPE are normalized, then $R_+$ = Reward+RPE and $R_-$ = Reward−RPE are their principal components. Furthermore, for positive correlations $r > 0$, the first principal component is $R_+$.

## Correlation and orthogonalization for EEG analysis

Novelty is nearly decorrelated from Surprise and NPE, but Reward and RPE are highly correlated with each other and also correlated with Novelty and NPE (S6 FigA). PCA on the variables Reward and RPE yields new decorrelated variables $R_+$ and $R_-$ (Section PCA over Reward and RPE). After projecting Surprise, Novelty, and NPE on the space orthogonal to $R_+$ and $R_-$ (S6 FigB), all five variables are decorrelated (S6 FigC, left part). Nevertheless, the new variables Surprise$_\perp$, Novelty$_\perp$, and NPE$_\perp$ remain very similar to the original variables Surprise, Novelty, and NPE as indicated by correlations equal to or above 0.89 (S6 FigC, right part).

The regression analysis without PCA and without orthogonalization (S7 Fig) yields results quite similar to the one with PCA and with orthogonalization (Fig 10 in the main text); the main difference is that the significant positive correlation of a reward-related variable ($R_+$) with the EEG amplitude in the 3rd time-window disappears. The reason is the very high correlation between Reward and RPE (S6 FigA), which leads to imprecise estimation of regression coefficients (compare S7 FigB and S7 FigC with Fig 10B and Fig 10C in the main text). Please note that since the regressors in S7 Fig are a linear combination of the regressors in Fig 10, the adjusted R-squared is the same for both analyses (compare S7 FigA with Fig 10A in the main text).
