# Supplementary material for: Novelty is not surprise: Human exploratory and adaptive behavior in sequential decision-making
Source: PLoS Comput Biol. 2021 Jun 3;17(6):e1009070. doi: 10.1371/journal.pcbi.1009070 (PMC8205159; doi:10.1371/journal.pcbi.1009070)
Supplement: S3 Text — (PDF) [file pcbi.1009070.s003.pdf]

Supplementary Information S3 Text: Fitted parameters and parameter-recovery

He A. Xu, Alireza Modirshanechi*, Marco P. Lehmann, Wulfram Gerstner, Michael H. Herzog

* alireza.modirshanechi@epfl.ch

Fitted Parameters

The optimal parameters after fitting the SurNoR model to behavior are summarized in S1 Table (corresponding to light green lines in S4 Fig). The reported error for each parameter is the maximum of its standard deviation approximated by Laplace approximation [1] and the optimization precision. Laplace approximation was done for each dimension separately, i.e. the covariance matrix was assumed to be diagonal to avoid the problems arising from approximation of the full Hessian matrix in high-dimensional spaces. Therefore, the reported errors can be seen as lower bounds for the real errors.

Simulated data, model recovery, and parameter recovery

The summary of the analyses of the data of two sets of 12 simulated participants (different from the one shown in Fig 7 in the main text) is shown in S2 Fig and S3 Fig. While we observe some variabilities in data generated by different random seeds, the main aspects of participants' behavior are captured by the model (Fig 7 in the main text, S2 Fig, and S3 Fig). The true model was successfully recovered in all 3 different cases (Fig 8 in the main text).

To check whether parameters of SurNoR are recoverable in our experimental paradigm, we fitted SurNoR to the three sets of 12 simulated participants (corresponding to the data shown in Fig 7 in the main text, S2 Fig, and S3 Fig). The recovered parameters are shown in the log-likelihood landscape in S4 Fig. The true parameters were successfully recovered with reasonable errors given the measured curvature of the log-likelihood function.

Robustness of model-variables in EEG analysis

In the EEG analysis in Fig 10 of the main text, we used model variables (Surprise, Novelty, NPE, Reward, and RPE) that were calculated from the SurNoR algorithm with one specific parameter choice. We wanted to check the robustness of these model variables with respect to changes in the parameters of SurNoR. Novelty and Reward are by definition independent of the SurNoR parameters. To check the robustness of the other variables, we used the 3 sets of recovered parameters (S4 Fig) and recalculated, for each of our 12 participants, the time course of Surprise, NPE, and RPE for each of the three recovered parameter sets. The model variables extracted given the recovered parameters were extremely highly correlated (> 0.97) with the model-variables extracted given the fitted parameters (S5 Fig).

References

1. MacKay DJ. Information theory, inference and learning algorithms. Cambridge university press; 2003.
