# Supplementary material for: Novelty is not surprise: Human exploratory and adaptive behavior in sequential decision-making
Source: PLoS Comput Biol. 2021 Jun 3;17(6):e1009070. doi: 10.1371/journal.pcbi.1009070 (PMC8205159; doi:10.1371/journal.pcbi.1009070)
Supplement: S4 Text — (PDF) [file pcbi.1009070.s004.pdf]

Supplementary Information S4 Text: The analysis of random exploration

He A. Xu, Alireza Modirshanechi*, Marco P. Lehmann, Wulfram Gerstner, Michael H. Herzog

* alireza.modirshanechi@epfl.ch

For any stationary policy (e.g., random choice), the sequence of states $\{S_1, S_2, ...\}$ forms a stationary Markov chain. Let us define the random variable $\mathcal{T}$ as the time of the 1st encounter of the goal state, i.e., $\mathcal{T}$ is the length of the 1st episode. We connect the expected number τ_i of actions to find the goal starting from state $S_1 = i$ (with $i \in \{1, ..., 10\}$) to the expected number of actions τ_j in the possible *next* states $S_2 = j$,

$$\tau_i = \mathbb{E}[\mathcal{T}|S_1 = i] = 1 + \sum_{j=1}^{10} p_{ij}\tau_j, \tag{1}$$

where p_{ij} is the probability of transitioning from state i to state j (dependent on the stationary policy), and we have already exploited that the goal state does not contribute in the sum because τ_G is by definition zero.

For a random policy (0.25 probability for each of the four actions) and the layout of the environment in Fig 1 in the main text, we find $\tau_{\text{trap}} := \tau_8 = \tau_9 = \tau_{10} = \tau_1 + 4$, because it takes on average 4 actions to leave the trap states. Similarly, from state 7, you have a probability of 1/4 to reach the goal in one step, but you can also remain in state 7 or go to one of the trap states. Evaluating all possibilities we arrive at

$$\begin{aligned} \tau_{\text{trap}} &= \tau_1 + 4, \\ \tau_{i+1} &= 3\tau_i - 2\tau_{\text{trap}} - 4 \ \text{ for } \ i \in \{1, ..., 6\}, \\ \tau_7 &= \frac{4}{3} + \frac{2}{3}\tau_{\text{trap}}. \end{aligned} \tag{2}$$

By solving this set of linear equations, we find

$$\begin{aligned} &\tau_1 = 13116 \ , \ \tau_2 = 13104 \ , \ \tau_3 = 13068 \ , \ \tau_4 = 12960 \\ &\tau_5 = 12636 \ , \ \tau_6 = 11664 \ , \ \tau_7 = 8748 \\ &\tau_8 = \tau_9 = \tau_{10} = \tau_{\text{trap}} = 13120. \end{aligned} \tag{3}$$

The results of calculation show that, starting from state 6 (which is the starting state of the first episode in our experiments), it takes on average more than 10000 actions to find the goal with a random policy.
