# Supplementary material for: Novelty is not surprise: Human exploratory and adaptive behavior in sequential decision-making
Source: PLoS Comput Biol. 2021 Jun 3;17(6):e1009070. doi: 10.1371/journal.pcbi.1009070 (PMC8205159; doi:10.1371/journal.pcbi.1009070)
Supplement: S5 Text — (PDF) [file pcbi.1009070.s005.pdf]

Supplementary Information S5 Text: Precise statement of the prediction in ‘Discussion’

He A. Xu, Alireza Modirshanechi*, Marco P. Lehmann, Wulfram Gerstner, Michael H. Herzog

* alireza.modirshanechi@epfl.ch

Consider an extended version of our environment in Fig A which includes a new (and not necessarily finite) set of states (i.e., the purple states in Fig A) that can be accessed from state 4 in the middle of the direct path to the goal. Assume that a participant has found the goal state G at the end of the first episode. In episodes 2 to 5 two different situations may arise. (i) If participants believe that the yellow goal in Fig A is the only (or the most) rewarding state in the environment, then they should ideally stop exploration as soon as they have found the goal and go straight to the goal in subsequent episodes. (ii) If participants wonder whether there may exist another state with a higher value of reward than state G, then they will spend a large amount of time in novelty-rich states like the purple states in Fig A. Our prediction, based on the SurNoR model presented in the main text, is that both situations can be observed in the behavioral data and that the difference depends on the prior knowledge given to the participant about the environment before the start of the experiment.

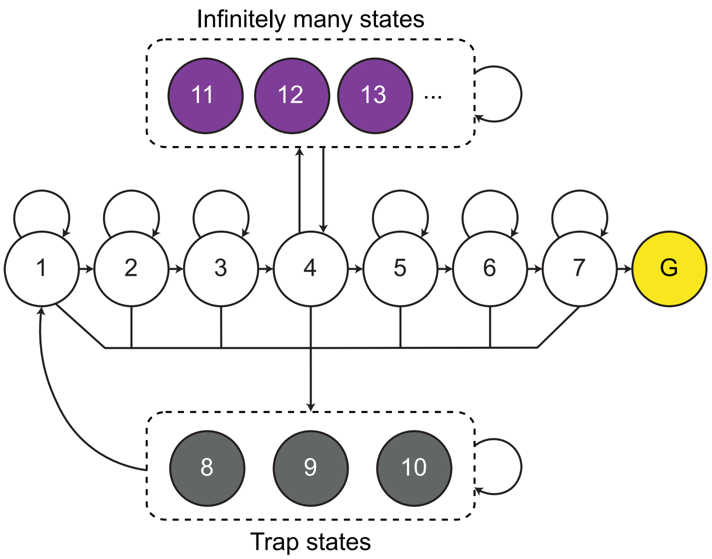

Fig A. An example of the extended version of our environment mentioned in the ‘Discussion’ section of the main text. The existence of a set novelty-rich states may distract participants from exploiting the reward at the goal in the episodes after the 1st episode.

The environment of Fig A also provides a critical test for alternative algorithms of SurNoR. Importantly, in the SurNoR model, information on novelty and external reward are summarized in two separate set of Q-values. Consider an alternative model where the novelty is treated as an internal reward and is added to the external reward

in a *single* set of Q-values. This is equivalent to adding Q-values of novelty and reward with a *fixed* factor β_N (see S1 Text).

Since the novelty of the purple states is constantly increasing, no matter the values of β and ω in S1 Text, any fixed and non-zero value of β_N (see S1 Text) will eventually drive the agent back towards novelty-seeking and hence exploration of the purple states. This statement holds for non-deterministic model-free, model-based, or hybrid models.

A straightforward way to avoid being distracted from exploiting the external reward is to stop seeking novelty after finding the goal for the first time. This is done in the SurNoR algorithm by reducing β_N, i.e., by reducing the relative importance of novelty.
