# Supplementary material for: Novelty is not surprise: Human exploratory and adaptive behavior in sequential decision-making
Source: PLoS Comput Biol. 2021 Jun 3;17(6):e1009070. doi: 10.1371/journal.pcbi.1009070 (PMC8205159; doi:10.1371/journal.pcbi.1009070)
Supplement: S6 Text — (PDF) [file pcbi.1009070.s006.pdf]

Supplementary Information S6 Text: Qualitative differences between model-based (MB) and model-free (MF) branches of SurNoR

He A. Xu, Alireza Modirshanechi*, Marco P. Lehmann, Wulfram Gerstner, Michael H. Herzog

* alireza.modirshanechi@epfl.ch

The difference in log-evidence of SurNoR and MF+N+S (Fig 5A in the main text) shows that both branches, MF and MB, are need for explaining behavior, although the participants' decisions are dominated by the MF action choices (Fig 6A4 in the main text). We wanted to find out at which point in the experiment the policy of MB is different from MF.

To do so, we focus on the 1st episode of block 1 and analyze the MB and MF branches of SurNoR with its parameters fitted to behavior (S1 Table). We analyze below two different situations where MB and MF have different preferences and show that in both cases a hybrid policy explains the behavior better than either MF or MB separately. Note that our analyses are based on the specific set of fitted parameters.

After the 1st failure

Consider an agent and assume that during the first visit of state 1 it has chosen one of the bad actions. We wondered which action the agent chooses the next time it visits state 1: Does it repeat its last action or choose another action? We, therefore, analyzed the behavior of participants, SurNoR, the MB branch of SurNoR, and the MF branch of SurNoR in this situation for state 1, 2, and 3 (Fig AA). In all three states, while the MB branch of SurNoR favors changing the action (and exploring the ones not chosen before), the MF branch of SurNoR favors repeating the same action as chosen the first time. SurNoR combines the two and prefers changing the action, which is consistent with the behavior of participants.

The reason that the MB branch prefers a new action is that it is aware of the existence of many other states that are more novel than any of the trap states, and hence, it assigns a larger novelty value $Q_{\mathrm{MB,N}}$ to unexplored actions than the previously chosen one. On the other hand, because the initial value Q_{N0} of MF Q-values is very small (S1 Table and S4 Fig), the first encounter of a trap state increases the MF novelty value $Q_{\mathrm{MF,N}}$ of the chosen state-action pair. Taken together, the MB branch literally plans to find more novel states while the MF branch follows what have been internally rewarding before. Hence, the MB branch is important to correctly guide action choices after the first failure.

After the nth success

Consider an agent and assume that it has chosen n times the good action in state 1. We wondered which action the agent chooses the next time it visits state 1: Does it further repeat the good action or choose another action? We, therefore, analyzed the behavior of participants, SurNoR, the MB branch of SurNoR, and the MF branch of SurNoR in this situation for n equal to 2, 6, and 9 (Fig AB). As n increases, the MF branch of

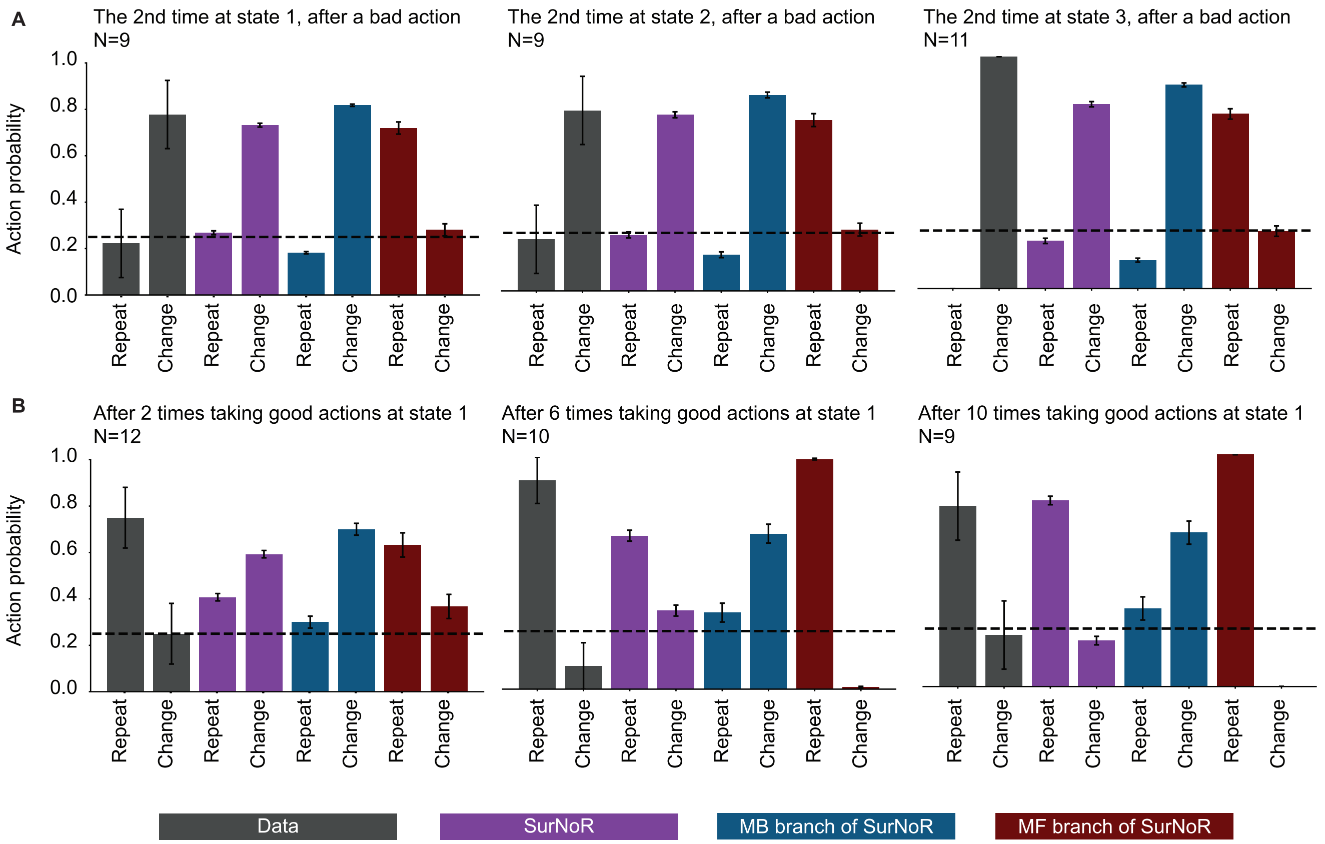

Fig A. The MB and MF branches of SurNoR have different preferences in different situations. Participants behave more MB in some situations and more MF in the others, and a hybrid policy can capture their behavior in both situations. **A.** Average probability of repeating the previous action or changing to another one in states 1, 2, and 3, with the condition that the 1st time the agent took the bad action. Error bars show the standard error of the mean. **B.** Average probability of repeating the good action or changing to another one after 2, 6, and 9 times taking the good action in state 1. Error bars show the standard error of the mean.

SurNoR increases its preference for staying with the good action, while the MB branch of SurNoR still favors exploring the environment (although with less confidence compared to the case discussed above, Fig AA). SurNoR combines the two, and, as n increases, gets closer to the MF preferences and to the participants' behavior.

In summary, in the 1st situation, participants are more MB, while in the 2nd situation, they are more MF. The take-home message is that the hybrid policy flexibly combines the two and explains the participants' behavior in both situations.
