# Supplementary material for: Novelty is not surprise: Human exploratory and adaptive behavior in sequential decision-making
Source: PLoS Comput Biol. 2021 Jun 3;17(6):e1009070. doi: 10.1371/journal.pcbi.1009070 (PMC8205159; doi:10.1371/journal.pcbi.1009070)
Supplement: Attachment — Submitted filename: Response_letter.pdf [file pcbi.1009070.s015.pdf]

Response to the Reviewers

April 27, 2021

We thank the editor and all reviewers for their positive, constructive, and helpful feedback! We have significantly revised our manuscript following comments and suggestions of the editor and reviewers. Please find our responses below.

Editor

Dear Mr. Modirshanechi,

Thank you very much for submitting your manuscript "Novelty is not Surprise: Human exploratory and adaptive behavior in sequential decision-making" for consideration at PLOS Computational Biology.

As with all papers reviewed by the journal, your manuscript was reviewed by members of the editorial board and by several independent reviewers. In light of the reviews (below this email), we would like to invite the resubmission of a significantly-revised version that takes into account the reviewers' comments.

I would like to amplify a few points made by the reviewers:

- The evidence for the winning model comes mainly from model comparisons. It is important to show through model-agnostic analysis and predictive checks what exactly the winning model is capturing which the other models are not.

Response 1. Following your suggestions, we conducted several model-agnostic analyses to investigate what aspects of the participants' behavior are captured by the winning algorithm (SurNoR). The results are reported in the new Figures 6B and 7 in the main text, and the new Figures S3, S4, and S8 in Supplementary Material. The new results show the benefits of using surprise-modulation for learning (the new Figures 6B, 7A, 7D, S3A, S3D, S4A, and S4D), novelty-seeking for exploration (the new Figures 7, S3, and S4), and the hybrid policy for action-selection (the new Fig. S8) more explicitly and in a more intuitive and comprehensive manner.

- 12 subjects is far too few for this kind of study, particularly involving EEG, which has a relatively low signal-to-noise ratio. Given the current circumstance, I won't ask you to collect more data (though that would of course be welcome), but you need to demonstrate using model recovery that your experimental setup is in principle capable of supporting your conclusions.

Response 2. We used model-recovery and showed that when the data is generated by SurNoR

(with its parameters fitted to behavior), the true model can be successfully recovered given the data of 12 simulated participants - by using the same model-selection procedure as we used for the real data (the new Fig. 8). We also showed that the true parameters were recoverable with reasonable errors (the new Fig. S5) and our model-variables (e.g. RPE) were very robust with respect to changes in the parameters (the new Fig. S6). See Response 5 for more details.

- Some of the models are very complicated and have many free parameters. This is not a problem in general, but my concern is that you are drawing rather strong conclusions about your model space from a specific task that is very simple compared to the complexity of the models. There's no way for your task to tease apart the roles of all the different parts of the models. It would build confidence that you're not overfitting if you were able to show that the winning model could also capture data from other sequential decision tasks. There are multiple publicly available data sets that you could analyze. However, I will not make this a requirement of the revision if you can demonstrate at least through simulation that the model produces sensible behavior on other tasks.

Response 3. This is a very interesting point, and we are grateful that you have brought it up. However, there are 2 important points:

First, our experiment is quite complex and is designed specifically to capture all three exploratory (episode 1 of block 1), adaptive (episode 1 of block 2), and exploitative (episodes 2-5 of blocks 1 and 2) aspects of behavior. Moreover, to build our model space, we have taken a modular approach (now explained more explicitly in the text, see Response 9) and combined different mechanisms for explaining these three aspects of behavior. Therefore, we can confidently say that the experiment *is* complex enough to "tease apart the roles of all the different parts of the models" - especially given our new model-agnostic analyses (see Response 1).

Second, indeed, most of the tasks with publicly available data-sets (e.g., variants of 2-step tasks or variants of bandit tasks) are way simpler than our experiment; moreover, most of the models that have been shown to be successful in explaining human behavior in those tasks (e.g., a simple hybrid algorithm or a simple Bayesian change-point detection algorithm) are nested in SurNoR. Therefore, if we do not fix the parameters, then demonstrating "through simulation that [SurNoR] produces sensible behavior on other tasks" is a trivial task, since we know there exists subsets of parameters for which SurNoR reduces to the models that have been previously investigated on those tasks. On the other hand, fixing the parameters of SurNoR at their values fitted to our experiment and then simulating it on other tasks does not make sense, because of the obvious reason that many parameters are dependent on the features and structure of environments and participants' inductive biases. For example, if participants know (or infer from obvious cues) the general model of the environment (e.g., visual cues that suggest a two-dimensional layout of states in an environment that is indeed consistent with such cues), then they might act more model-based - see for example Da Silva and Hare (2020). As another example, if the participants infer that the environment is less volatile (i.e., there is a smaller probability for an abrupt change), then, in principle, the effect of surprise-modulation must decrease, since a surprising event is more likely to be due to pure randomness instead of an abrupt change of the environment (Nassar, et al. 2010, 2012 and Liakoni, et al. 2021). Therefore, to be able to use the same set of parameters, one needs to add yet another level of hierarchy in order to be able to model the prior assumptions and inductive biases (similar to Wilson, et al. 2010 in Neural Computation), which is out of the scope of our current paper.

To make sure that we do not overclaim, we added the following paragraphs to the Discussion

section and explicitly mentioned the limitations of our conclusions:

> Our results extend findings that humans use hybrid policies in two-stage decision tasks [5, 6] to the case of deep sequential decision tasks in the presence of abrupt changes. In general, the balance between model-free and model-based behaviors depends on multiple aspects and features of the task that humans are dealing with [3, 5, 70, 71]. For example, we observe that the model-based branch becomes more important when participants explore the environment to find the goal state, although the participants' behavior in our environment is always dominated by model-free action choices (Fig. 6A4). Moreover, the dominance of model-free behavior in such deep tasks does not exclude that humans used model-based planning in shallow tasks that are easily comprehensible thanks to a spatial arrangement of states or explicit instructions [72].
>
> An interesting direction for future studies is to combine surprise modulation with more abstract model building algorithms, e.g., for learning the structure of neighborhood relations of an environment in the form of a graph [73, 74]. Such algorithms may explain the slight difference between the participants' adaptive behavior and SurNoR's predictions (Fig. 6B).

and

> In general, it has been shown that exploration in humans can have more than one drive [77] and that participants' desire for seeking novel events depends on their goal, inductive biases, and assumptions about their environment [9, 15, 37]. In situations like our experimental paradigm where participants are sure that there exists a rewarding state but do not know how to reach it, seeking novelty and exploring the parts of the environment that have been less visited are natural ways to search for the rewarding state; however, if, for example, participants were asked to find the most accurate map of the environment, then uncertainty-seeking might be a more reasonable way to solve the task. In addition, the presence of trap states in our environment makes novelty an internally rewarding signal that helps participants to avoid 'traps'. We do not claim that novelty is always the only drive of exploration; rather, we believe that our results show that for a class of tasks similar to ours where the goal is to search for a rewarding state and novelty is a relevant signal, humans use a novelty-seeking strategy for exploration. Following the idea of information search in active sampling [78, 79], we speculate that whether novelty is an informative cue (e.g., about the location of the goal) or not must be itself inferred by participants through the exploration procedure. From a different but similar perspective, we speculate that it is possible to take a normative approach and, by defining a function of curiosity [15, 37], show that novelty-seeking is the optimal or a close to optimal way to search for reward in a class of environments. Formulating and testing such hypotheses is an interesting direction for future studies.

- The definition of "novelty" is problematic, since (as pointed out by one of the reviewers) it implies that a frequently experienced stimulus could be defined as novel as long as other stimuli are even more frequently experienced. This defies common sense. Essentially this is a terminological issue.

Response 4. Thanks for pointing this out. Indeed the word 'novelty' has different meanings. Therefore, in the section that we define novelty for the 1st time, we now write:

> In the Oxford English Dictionary [45], novelty is defined as 'the quality or state of being new, original, or unusual'. Here, our focus is on the quality of being 'unusual', and by saying that a state is novel, we mean that it has not been encountered often, i.e., it is not 'usual' to encounter this state. We, therefore, assume that (i) the novelty of a state s at time t is a decreasing function of the number $C_s^{(t)}$ of encounters of state s until time t, e.g., a state that has been encountered 5 times is less novel than a state that has been encountered only once. Moreover, we assume that (ii) a state s that has been encountered for example $C_s^{(t)} = 5$ times within a total of $t = 5$ trials is less novel compared to a state s' that has been encountered for example $C_{s'}^{(t')} = 5$ times within a total of $t' = 500$ trials. Following assumptions (i) and (ii), we define the novelty of a state s as a decreasing function of the observation frequency
>
> $$p_N^{(t)}(s) = \frac{C_s^{(t)} + 1}{\left(\sum_{s'} C_{s'}^{(t)}\right) + 11}.$$

In the same section, we also mentioned your concern and added:

> Since our novelty measure depends on the frequencies (relative counts, $p_N^{(t)}(s)$ in Eq. 1) rather than the raw counts ($C_s^{(t)}$), one may also interpret $N^{(t)}(s)$ as a measure of 'relative novelty' or 'rareness'. See Discussion for the relation of our measure of novelty to other measures.

We then added a rather long discussion about our definition of novelty and its links to other definitions in neuroscience to the Discussion section:

> In the SurNoR model, surprise measures how *unexpected* the next state is according to an acquired world-model *conditioned* on the current state and the chosen action; in contrast, novelty measures how *infrequent* the next state has been, *independent* of our expectations derived from a world- model. More precisely, in our formulation (Eq. 2 and Eq. 4), surprise and novelty have two essential differences: The first difference is that while surprise is assigned to transitions, novelty is assigned to states. If this was the only difference between novelty and surprise, one could argue that surprise and novelty are essentially the same, while one measures the infrequency of transitions and the other the infrequency of states. However, the second and more important difference is that novelty uses the exact number of encounters of each state (Eq. 1) to measure how infrequent that state has been, while surprise uses the surprise-modulated pseudo-counts (Eq. 3) computed by the world- model to measure how unexpected a transition is. As a consequence, if there is a sudden change in the environment, then an expected transition can rapidly become surprising, but a long time is needed for a state that has been encountered frequently to become novel again. This is consistent with ideas that novelty is more related to memory-recall and surprise is more related to predictions [35]. Moreover, these ideas are also supported by recent findings showing that the

brain estimates the frequency of stimuli over a much longer time-scale than the transition probabilities [22].

[...]

Measures of novelty can also be divided into two subgroups [35]: the ones that are 'memory-based' and the ones that represent 'statistical outliers'. Memory-based novelty measures focus on whether an event already exists in the memory or not [65, 66]. Our measure of novelty (Eq. 2) belongs to the 2nd group that considers an event as novel if it has 'a low estimated probability of occurrence' [35]. Many measures of novelty that belong to this group simply consider novelty to be a decreasing function of the number of occurrences [9, 15, 17]; in contrast, our measure of novelty is a decreasing function of the frequency of occurrence (Eq. 1), and because of this reason one may refer to it as 'relative novelty' or a measure of 'rareness'.

We cannot make any decision about publication until we have seen the revised manuscript and your response to the reviewers' comments. Your revised manuscript is also likely to be sent to reviewers for further evaluation.

[...]

Sincerely,

Samuel J. Gershman
Deputy Editor
PLOS Computational Biology

Reviewer #1

In the current study, individuals completed a sequential decision task with two unique features. First, the task included 'trap' trials, creating more visits in some states than others. Second, at the second half of the session, two states were swapped, creating transitions that deviated from the transition probability the individuals learned in the first part of the experiment. These two elements in the task design are used to test whether individuals are using 'state-novelty' (i.e., related to number of visits in a state), and 'ransition-supervise' (related to getting to an unexpected state) to direct their actions. The authors describe in detail a hybrid model where novelty-seeking serves as a motivational goal, and transition-surprise modulates learning-rates. The authors claim that a hybrid model with model-based model-free, novelty and reward learning overcomes as models. The authors further argue using EEG data for distinct correlation between these two elements (i.e., 'state-novelty' and 'transition-surprise') with neural activity.

I would like to thank the authors for their hard work, which I believe is very interesting and provide further insights into action selection in humans. I think the manuscript enjoys both a details computational section, empirical model comparison and a section with neuronal activity correlations. In that sense, I believe the manuscript is well within the aims and scope of the current journal. I for have one major comment regarding model-selection that I will detail below. I hope this will serve the authors in improving their work, and I would like wish good luck and further fruitful studies.

Yours,
Nitzan Shahar
Sagol school of Neuroscience
Tel-Aviv University

Major Comment:

My major concern regards the fact that the model comparison includes a large amount of models (13 models), the winning model has a large amount of parameters (18 parameters), yet the data set is small (12 participants with 250 trials each). I would not a priori say it is impossible to make conclusions based on such setting, but I think we need to make sure the model selection method is justified, and that both the winning model and the parameter estimates are recoverable in this setting. The authors pooled data across three groups of subjects (4 subjects each), and used cross-validated maximum log-likelihood and protected exceedance probabilities for model selection. I think the model selection evidence will be much more compelling if the authors could show that: (a) the parameters of the winning model (which other than one model have the largest number of parameters in the model space) can be recovered in a simulation with the same number of subjects and trials. (b) show that the true model can be accurately recovered in this setting and with the same number of subjects, trials and pooling method. That is, simulate data and show we can accurately identify which model generated the data from all the relevant models. If the authors have in mind any other way to address parameter and model recoverability, that would be great as well.

Response 5. We are very grateful for this comment and believe that adding this analysis made our conclusions much more promising.

Following your suggestion, we generated data for 3 sets (with different random seeds) of 12 simulated participants using SurNoR with its parameters fitted to behavioral data (the same

set of parameters as used for EEG analysis). The summary of the generated data is shown in the new Fig. 7 of the main text and the new Figures S3 and S4 of Supplementary Material. Since the number of trials is itself a random variable, it was not possible for us to follow your suggestion to keep it exactly the same as the number of trials of human participants. However, the model captures the behavior well enough, and the number of trials of simulated participants was roughly in the same range as the numbers of trials of human participants (the new Figures 7A, S3A, and S4A).

(a) We fitted the parameters of SurNoR separately to each of the 3 sets of simulated participants - by applying the same optimization technique that we had used for fitting SurNoR parameters to behavioral data. The results are shown in the new Fig. S5 of Supplementary Material and show that the true parameters were successfully recovered with reasonable errors (compared to the curvature of the log-likelihood function). We also showed that the amount of error introduced in recovery of parameters does not change the model variables that are used for correlation with the EEG data (e.g., RPE and NPE) much - see the new Fig. S6 of Supplementary Material.

(b) We used the same model-selection approach (the same pooling, number of folds, optimization method, etc.) and showed that SurNoR can be successfully recovered given the data of any of the 3 sets of the simulated participants (the new Fig. 8). The only difference between the model-recovery procedure and the model-selection procedure was that, for model-recovery, we included only the models that had turned out to be the best ones for the real data (Fig. 5) and allowed us to test the main three components (hybrid policy, novelty-seeking, and surprise modulation) of SurNoR: MF+S+N, Hyb+S+U, and Hyb+N. We made this choice because of the limited computational resources and for the sake of saving time - since for each random seed, the model-recovery took between 5 to 7 days, depending on the number of trials.

Minor comments:

Line 495: The weight of $Q^{(t+1)}_{MF,N}$ and $Q^{(t+1)}_{MB,N}$ is non-zero only in the 1st episodes of blocks 1 and 2. If I read this correctly, it means the author guides the model as to where 'novelty matters', affectively muting novelty prediction errors in blocks where novelty was assumed lower by design. I think it would be better to explain what happens when this is not done and whether the model comparison results are changed when novelty prediction errors are not muted in the whole task. I would expect the model to be able to win even with stricter conditions where novelty prediction errors take part across the whole task.

Response 6. We first need to clarify that we never muted novelty prediction errors (NPE), and the NPE signals were used by SurNoR in all episodes of both blocks to update the novelty Q-values. However, as correctly pointed out by you, we turned off the effect of novelty Q-values on policy in episodes 2-5 of blocks 1 and 2. We tested your suggestion, and it turned out to be correct. Here is what we added to Methods:

> The weight of $Q^{(t+1)}_{\mathrm{MF,N}}$ and $Q^{(t+1)}_{\mathrm{MB,N}}$ is non-zero only in the 1st episodes of blocks 1 and 2 to reduce number of parameters and make the model simpler. We tested the version with an additional free parameter for the weights of $Q^{(t+1)}_{\mathrm{MF,N}}$ and $Q^{(t+1)}_{\mathrm{MB,N}}$ in episodes 2-5 of blocks 1 and 2, but we did not find any significant improvement in the fit (difference in log-evidence $= 15 \pm 13$).

Line 255: We find that the model-free branch dominates the actions. Thus the world-model

is of secondary importance for action selection and is mainly used to detect surprising events. Given this conclusion, I would assume that the advantage of a hybrid model over a model-free only model comes mostly from few trials in the first episode of the second block, where surprise is manipulated experimentally. Yet the SurNoR model seems to win even when only behavior from the first block is used (Figure 4A). Why is that? If really the whole purpose of the model-based branch is to direct learning rate in these cases, wouldn't a simpler algorithm manipulating model-free learning rates (e.g., Pearce-Hall associability) lead to similar results with less parameters? I think the authors should at least mention these options, even if they are not fitted to the data directly.

Response 7. Please note that MF+N+S has all elements of SurNoR (including a world-model and a surprise-modulated learning rate) except for the model-based planning. The fact that MF+N+S is outperformed by SurNoR (Fig. 5) is clear evidence that the effect of a hybrid policy in explaining behavior is *significant*. What we show in Fig. 6 is that this effect, although *significant*, is *small* ("of secondary importance"), and that is what we mean by what you quoted. To be sure that we make it clear that we do not claim that humans are model-free, we added the following paragraph to the Discussion section:

> Our results extend findings that humans use hybrid policies in two-stage decision tasks [5, 6] to the case of deep sequential decision tasks in the presence of abrupt changes. In general, the balance between model-free and model-based behaviors depends on multiple aspects and features of the task that humans are dealing with [3, 5, 70, 71]. For example, we observe that the model-based branch becomes more important when participants explore the environment to find the goal state, although the participants' behavior in our environment is always dominated by model-free action choices (Fig. 6A4). Moreover, the dominance of model-free behavior in such deep tasks does not exclude that humans used model-based planning in shallow tasks that are easily comprehensible thanks to a spatial arrangement of states or explicit instructions [72].

To address your question why SurNoR explains behavior better even in the 1st episode of block 1, and to make the benefits of a hybrid policy more explicit, we analyzed two situations in the 1st episode of block 1 where the model-free and the model-based branches of SurNoR have different preferences over actions. The results are shown in the new Fig. S8 in Supplementary Material and are discussed in section "Qualitative differences between model-based (MB) and model-free (MF) branches of SurNoR" in Supplementary Material. There we show:

> The MB and MF branches of SurNoR have different preferences in different situations. Participants behave more MB in some situations and more MF in the others, and a hybrid policy can capture their behavior in both situations.

See the section and the corresponding figure for full explanation. In the main text, we point to this section when we discuss the relative importance of the MF and the MB branches of SurNoR:

> Since SurNoR combines in its hybrid action selection policy a model-free with a model-based component (Fig. 6A2), we first wanted to analyze the relative importance of each of the two components in explaining the action choices of participants; see Supplementary Material for a qualitative comparison of these two components.

Lines 72 – 79: These lines are important- but very hard to follow. Please try to explain what you mean by episode and block, and how is it possible that individuals performed different episodes without "knowing" they started from the beginning (line 72)? It took this reader a few repetitions until I understood the design.

Response 8. We re-wrote these lines:

> The experiment was organized in 10 episodes, i.e., it ended after the 10th time that participants found the goal state. Unknown to the participants, we divided these 10 episodes into 2 blocks of 5 episodes each; we refer to the first 5 episodes as block 1 and to the second 5 episodes as block 2. During the 1st episode of block 1, participants took between 34 and 214 actions (mean 118 and std 54) until they arrived at the goal (Fig. 1C). They then continued for another 4 episodes, each time starting in a new initial state. The initial state for each episode was chosen randomly, but it was kept fixed across participants. After the 1st episode, participants had learnt to reach the goal in less than 20 steps (episodes 2 to 5 in Fig. 1C). After the end of the 5th episode (the end of block 1), two states (state 3 and 7 in Fig. 1D) were swapped, without announcing it to the participants. Participants continued for another 5 episodes with the novel layout of the environment (2nd block, Fig. 1D).

Reviewer #2

This is a very intriguing paper on how humans explore and learn a complex state space in search for reward. The authors developed a complicated sequential decision-making task, in which the states are connected in a complicated and recurring way, also giving rise to "trap" states, which move them back to the beginning. Without a generative model, this state space is hard to learn and lots of transitions need to be explored in order to find n efficient way to the reward. This is shown by the very large number of steps taken in the first episode of every block. The authors then proceed to develop and novel reinforcement learning model that includes and estimate of the novelty and surprise (defined in a precise mathematical way) and they undertake a comprehensive model selection procedure to conclude that there new model outperforms all the different alternative (and mostly degenerate versions) of their model. They also find an unexpected result, namely that humans do not seem to rely on their world model that they build as they are learning about the state space - as demonstrated by the relative importance of MF over MB learning.

The paper is well-written and suited for the PLoS CB readership. I think presentation of the different model variants in the exhaustive model comparison could be somewhat improved, if the modular nature of (a) MB/MF/Hyb, (b) S/noS, (c) OI/U/N and their systematic combination into different model variants could be made a bit more explicit.

Response 9. We followed your suggestion and explained the differences between our alternative algorithms with more focus on their modular structure:

> SurNoR has three main components: (i) action selection by Hybrid policy, (ii) exploration by novelty-seeking, and (iii) learning by surprise-modulation. To test our hypothesis, and to test whether all three components of SurNoR are necessary for explaining behavior or whether a simpler or an alternative model would have the same explanatory power, we compared SurNoR with 11 alternative algorithms plus a null algorithm based on a random choice (RC) of actions (Fig. 5A). Three out of 11 algorithms use a hybrid policy (+Hyb), five use novelty-seeking (+N), and seven use surprise-modulation (+S).
>
> Alternatives for (i) action selection were pure model-based (MB; 4 out of 11 algorithms) and pure model-free (MF; 4 out of 11) policies. Note that we allow for the possibility that MF algorithms are equipped with a world-model for computation of surprise but do not use this world-model for action-planning. As alternatives for (ii) exploration strategy, we used optimistic initialization (+OI; 3 out of 11) [19] and uncertainty (surprise) seeking [42, 43] (+U; 3 out of 11); see below for more explanations and Supplementary Material for details. Finally, as an alternative to surprise modulation, we used constant learning rates for learning the world-model and model-free Q-values [22, 23, 49, 50] (all algorithms without +S; 4 out of 11). For the details of the alternative algorithms see Supplementary Material.

Furthermore, I have two related suggestions for improvement of the paper.

1. The (model-free) analysis of the highly interesting exploration phase of the first episode in each block strikes me a really sparse. I only noticed the number of step take in each episode as a model-free measure of these data. However, it would be much more informative (and actually crucial to my second suggestion) to develop a few indices that would characterize the

explorative behavior in this stage. For instance, on could think of the (mean) step number, after which the subjects have learned to avoid one of all of the trap states. Or the step number, after which they have learned to avoid the "recurring" action, which brings then back to the current state. Or one could define a kind of "exploration-exploitation index" fro each subject and each state, which ist the number of exploratory actions taken in each state before they settle on an exploitative (and henceforth constant) policy in each state. I am sure there are more indices that one could think of to describe the explorative behavior in this crucial first episode.

Response 10. This was a great suggestion. We defined two indices for the model-free analysis of exploratory behavior in the 1st episodes. Our first index was the median number of actions participants took to escape the trap states each time they felt into them. For the second index, we first assigned 'progress' values to different types of actions at each progressing state: 1 to the good actions (the ones taking participants one step closer to the goal), 0.5 to the neutral actions (the ones making participants stay where they are), and -0.75 to the bad actions (the ones taking participants to the trap states); with this assignment, random choice exploration has 0 average progress. Then, we defined our second index as the average progress of participants each time visiting a progressing state. The results are reported in the new Fig. 2, with the following explanation in the text:

> Participants explored the environment to find the goal, but they received no intermediate reward or other sign of progress while doing this. If participants followed a purely random exploration (i.e., choosing each action with 1/4 probability), it would take them in the 1st episode of block 1 on average about 10^4 actions to find the goal, starting at any non-goal state (see Supplementary Material). This high number is an indication of the complexity and depth of our environment. Our results suggest that participants followed a non-random strategy for finding the goal (Fig. 1C). With increasing experience, the latency of escape from the trap states was reduced (Fig. 2A) and the good actions at progressing states were chosen with higher probability (Fig. 2B). It is important to note that these improvements were observed in the absence of any external feedback indicating progress and before the 1st encounter of the goal state.

and

> After the swap, participants continued escaping from the trap states (Fig. 2A) and choosing the good actions at the unchanged progressing states (states 1, 2, and 4 in Fig. 2C). Moreover, they rapidly adapted their behavior and found the new good actions at the swapped states (state 7 in Fig. 2C). Our results indicate that participants adapted their behavior to the new situation while exploiting the knowledge they had acquired before.

2. Having defined such indices above, one could use these as targets for an appropriate posterior predictive check of the SurNoR model. So far, I don't think the paper includes a proper PPC - in my reading of the paper, the accuracies shown in Fig 4C refer to the experimental data that was also used to fit the model, but the fitted model was not used to generate new synthetic data that can be analyzed w.r.t to the indices defined above. Such an analysis would also underline that the SurNoR model also generalizes beyond the experimental data.

Response 11. As you suggested, we added posterior predictive checks, looking at the indices defined above as well as the total number of actions in each episode. The results for 3 different random seeds are reported in the new Fig. 7 in the main text and the new Figures S3 and S4 in Supplementary Material. The following explanation is added to the text:

> Finally, to see if the SurNoR model captures, in addition to other aspects, also the exploratory behavior of participants (Fig. 1C and Fig. 2), we computed posterior predictive checks [54, 55]. To do so, we simulated SurNoR with its parameters fitted to behavior and generated data for 12 simulated participants; see Fig. 7 for one set of 12 simulated participants and see Supplementary Material for two other sets with different random seeds. Our results show that several important features of the behavior of participants are observed also in the behavior of the simulated participants: (i) They are faster in finding the goal in the first episode of block 2 than in the first episode of block 1 (Fig. 7A), (ii) they learn to escape the trap states and to choose the good action at progressing states in the 1st episode of block 1 (Fig. 7B and Fig. 7C), and (iii) after the swap, they continue choosing the same actions at unchanged states but rapidly unlearn previously learned actions at the swapped states (Fig. 7B and Fig. 7D).

Reviewer #3

Xu, Modirshanechi and colleagues present a novel computational framework in which novelty promotes exploration, while surprise adaptively adjusts the model's rate of adaptation. They show that this model, which uses novelty as a pseudo reward and surprise to adjust a local learning rate, offers a good explanation of human performance on a deep sequential learning task with sparse rewards. In addition, they identify signals correlated with the model's key variables in recorded EEG. The questions tackled by the authors; how do humans efficiently explore in complex environments with sparse reward information, and how does volatility influence learning and decision making in such an environment, are very much at the edges of both human and machine learning research, positioning this work as highly topical and of interest to a wide audience. However, there are a number of issues that limit the reach of the work presented here. Generally speaking, the computational model is exceptionally complex. I fear that while it offers an explanation of task performance presented here, it relies on several unconventional mechanisms that might obfuscate and limit its generalizability, calling into question its relevance to the more general fields of machine and human learning. I outline some of my specific concerns here:

- While it might be a touch trivial, the formulation of novelty does not appear to be consistent with how novelty is commonly framed. Rather, the model relies on a measure of relative stimulus frequency (how rare an image is relative to others). This formulation of novelty feels more akin to forms of surprise than novelty per se. As an example, according to equation 2, an agent that experienced 10000 observations in a world with 2 stimuli, with stimulus A presented 1000 times and B presented 9000 times, the model would treat stimulus A as highly novel despite having observed it 1000 times. Memory research would balk at this. While this is not an issue computationally, as tracking event rarity (be it an unexpected state transition, an unexpected reward, or an unexpected stimulus) likely offers a useful computational variable, it does have implications for the relevant background supporting the notion of 'novelty' driven exploration in animals and humans. For example, the authors cite previous work linking dopaminergic activity to the presentation of novel stimuli, but I know of no evidence suggesting that dopamine activity tracks relative stimulus frequency. Perhaps a more traditional formulation of novelty (e.g. a simple monotonically decaying function) could perform just as well?

Response 12. This is a very interesting point. Thanks for pointing it out. Indeed the word 'novelty' has different meanings. Therefore, in the section that we define novelty for the 1st time, we now write:

> In the Oxford English Dictionary [45], novelty is defined as 'the quality or state of being new, original, or unusual'. Here, our focus is on the quality of being 'unusual', and by saying that a state is novel, we mean that it has not been encountered often, i.e., it is not 'usual' to encounter this state. We, therefore, assume that (i) the novelty of a state s at time t is a decreasing function of the number $C_s^{(t)}$ of encounters of state s until time t, e.g., a state that has been encountered 5 times is less novel than a state that has been encountered only once. Moreover, we assume that (ii) a state s that has been encountered for example $C_s^{(t)} = 5$ times within a total of $t = 5$ trials is less novel compared to a state s' that has been encountered for example $C_{s'}^{(t')} = 5$ times within a total of $t' = 500$ trials. Following assumptions (i) and (ii), we define the novelty of a state s as a decreasing function of the observation

frequency

$$p_N^{(t)}(s) = \frac{C_s^{(t)} + 1}{\left(\sum_{s'} C_{s'}^{(t)}\right) + 11}.$$

In the same section, we also mentioned your concern and added:

> Since our novelty measure depends on the frequencies (relative counts, $p_N^{(t)}(s)$ in Eq. 1) rather than the raw counts ($C_s^{(t)}$), one may also interpret $N^{(t)}(s)$ as a measure of 'relative novelty' or 'rareness'. See Discussion for the relation of our measure of novelty to other measures.

We then added a rather long discussion about our definition of novelty and its links to other definitions in neuroscience to the Discussion section:

> In the SurNoR model, surprise measures how *unexpected* the next state is according to an acquired world-model *conditioned* on the current state and the chosen action; in contrast, novelty measures how *infrequent* the next state has been, *independent* of our expectations derived from a world- model. More precisely, in our formulation (Eq. 2 and Eq. 4), surprise and novelty have two essential differences: The first difference is that while surprise is assigned to transitions, novelty is assigned to states. If this was the only difference between novelty and surprise, one could argue that surprise and novelty are essentially the same, while one measures the infrequency of transitions and the other the infrequency of states. However, the second and more important difference is that novelty uses the exact number of encounters of each state (Eq. 1) to measure how infrequent that state has been, while surprise uses the surprise-modulated pseudo-counts (Eq. 3) computed by the world- model to measure how unexpected a transition is. As a consequence, if there is a sudden change in the environment, then an expected transition can rapidly become surprising, but a long time is needed for a state that has been encountered frequently to become novel again. This is consistent with ideas that novelty is more related to memory-recall and surprise is more related to predictions [35]. Moreover, these ideas are also supported by recent findings showing that the brain estimates the frequency of stimuli over a much longer time-scale than the transition probabilities [22].
>
> [...]
>
> Measures of novelty can also be divided into two subgroups [35]: the ones that are 'memory-based' and the ones that represent 'statistical outliers'. Memory-based novelty measures focus on whether an event already exists in the memory or not [65, 66]. Our measure of novelty (Eq. 2) belongs to the 2nd group that considers an event as novel if it has 'a low estimated probability of occurrence' [35]. Many measures of novelty that belong to this group simply consider novelty to be a decreasing function of the number of occurrences [9, 15, 17]; in contrast, our measure of novelty is a decreasing function of the frequency of occurrence (Eq. 1), and because of this reason one may refer to it as 'relative novelty' or a measure of 'rareness'.

- Behavioral adaptation is a function of both the learning rate and the magnitude of the prediction error signal. Transitionally, prediction errors offer a means of driving local adaptation

(where the prediction errors are large), while the learning rate offers a more global rate of adaptation. From what I understand, the authors use surprise (violation of model-transition expectations) to modulate learning locally at the source of surprise. Could the rapid adaptation at the start of block 2 not also be captured by a larger prediction error than what is offered by the model? What behaviour is captured specifically by a higher learning rate that could not be captured by larger prediction errors? Can a locally modulated learning rate and locally generated prediction error (which may not be accurately modelled) be robustly separated in this task?

Response 13. Our alternative algorithm Hyb+N is equivalent to SurNoR but with fixed learning rates (one for learning MF Q-values and one for learning MB world-model). While experiencing a surprising trial that, for example, brings an agent to one of the trap states while it has expected to go to another progressing state, the agent experiences a high surprise (and also a large state prediction error, SPE) and a high reward prediction error (RPE). If a high SPE and a high RPE with fixed learning rates were sufficient for explaining the adaptive behavior, then we would have seen the same explanatory power for Hyb+N and SurNoR, which is not the case according to our model comparison results (Fig. 5A). To make this difference between Hyb+N and SurNoR more explicit and comprehensive, we added a new analysis shown in the new Fig. 6B:

> [...] we compared the action choices of participants with those of SurNoR and the model Hyb+N (i.e., SurNoR without surprise-modulation) at the swapped states in the 1st and 2nd episode of block 2 (Fig. 6B). Our results show that the modulation of the learning rate by surprise in SurNoR is necessary to explain the rapid adaptation of participants after the switch of states.

Please note that the reasons behind why high prediction errors with a constant learning rate are not sufficient for learning in volatile environments has been discussed before both by ourselves in our earlier studies (Liakoni, et al. 2021; Faraji, et al. 2018) and also much 10 years ago by others in simpler situations (Nassar, et al. 2010, 2012).

- The model uses state frequency as a pseudo reward to guide exploration on episode 1. Relative to a hand-crafted pseudo reward function, it's refreshing to see a feature of the environment used in this way. However, I fear that a hand crafted pseudo reward has been exchanged for a hand-drafted environment where state frequency is confounded with task performance. Relative frequency is not predictive of reward in many, if not most environments. Does this mechanism for exploration extend to other environments, particularly where rarely encountered states are the only path to the goal (e.g. unique paths of different length that lead to the goal)?

Response 14. We agree with your point that such an exploration mechanism may not generalize to all other types of environments. To address this issue, we added a detailed discussion about the limitations of our results to the Discussion section:

> In general, it has been shown that exploration in humans can have more than one drive [77] and that participants' desire for seeking novel events depends on their goal, inductive biases, and assumptions about their environment [9, 15, 37]. In situations like our experimental paradigm where participants are sure that there exists a rewarding state but do not know how to reach it, seeking novelty and exploring the parts of the environment that have been less visited are natural ways

to search for the rewarding state; however, if, for example, participants were asked to find the most accurate map of the environment, then uncertainty-seeking might be a more reasonable way to solve the task. In addition, the presence of trap states in our environment makes novelty an internally rewarding signal that helps participants to avoid 'traps'. We do not claim that novelty is always the only drive of exploration; rather, we believe that our results show that for a class of tasks similar to ours where the goal is to search for a rewarding state and novelty is a relevant signal, humans use a novelty-seeking strategy for exploration. Following the idea of information search in active sampling [78, 79], we speculate that whether novelty is an informative cue (e.g., about the location of the goal) or not must be itself inferred by participants through the exploration procedure. From a different but similar perspective, we speculate that it is possible to take a normative approach and, by defining a function of curiosity [15, 37], show that novelty-seeking is the optimal or a close to optimal way to search for reward in a class of environments. Formulating and testing such hypotheses is an interesting direction for future studies.

- The cross-validation processes used to optimize free parameters and compare goodness of fit has both pros and cons. One chief benefit comes from the fact that the log-evidence quantifies how well parameter estimates generalize. However, this can be problematic when there are considerably individual differences in task performance as one individual may not in fact be well represented by others in the sample (as appears may be the case here, particularly in the critical first episodes of both blocks). Do model comparison results hold if parameters are optimized and penalized using other methods (more traditional gradient descent optimization and compared using measures like AIC or BIC)? That is, are the results reported here dependent on cross-validation in a relatively small sample?

Response 15. We think that your concern is in general valid. However, according to our posterior predictive checks, SurNoR with a fixed set of parameters can also capture the between-subject variability observed in the data (compare Figures 1 and 2 with Fig. 7 in the main text and Figures S3 and S4 in Supplementary Material). Therefore, the between-subject variability observed in the behavioral data is not necessarily due to a systematic difference between subjects; rather, it might be because of the essential randomness of action-selection. Therefore, to save time and because of the limited computational resources, we decided to not repeat our model selection. We added a sentence to Methods in order to point out that one could use other approximations of log-evidence:

> Similar to studies in economics and statistics [91, 92], we considered, for each participant and each algorithm, the cross-validated maximum log-likelihood (averaged over the 4 repetitions) as the log-evidence [93]. Similarly, the log-evidence could also be approximated by other measures like AIC or BIC [94], but cross-validation has been shown to have a more robust behavior [95].

- The model's exploration is explicitly turned off once the target image is found. The authors explain that exploration is no longer necessary once the goal is found. However, the task issued to participants was to find the shortest path to the target image. Thus, from the participant's perspective (since they don't know the task structure), simply finding the target image does not necessarily solve the task (e.g. there could be more efficient paths to the target image). As participants may continue to search for a shorter path in subsequent episodes,

explicitly dampening exploration in models being compared may unfairly hinder their ability to capture variance. Is it necessary that the pseudo-reward mechanism in the model be manually manipulated?

Response 16. We had put the weight of novelty-seeking equal to zero for episodes 2-5 of blocks 1 and 2 for the sake of keeping the model simpler and reducing the number of parameters. To address your point, we tested a variant of SurNoR with a preference for novelty also in episodes 2-5 of blocks 1 and 2; the results are reported in the Methods section:

> The weight of $Q^{(t+1)}_{\text{MF,N}}$ and $Q^{(t+1)}_{\text{MB,N}}$ is non-zero only in the 1st episodes of blocks 1 and 2 to reduce number of parameters and make the model simpler. We tested the version with an additional free parameter for the weights of $Q^{(t+1)}_{\text{MF,N}}$ and $Q^{(t+1)}_{\text{MB,N}}$ in episodes 2-5 of blocks 1 and 2, but we did not find any significant improvement in the fit (difference in log-evidence $= 15 \pm 13$).

- The model includes a wide array of variables. Are model parameters recoverable - that is - if the model generates a set choice using a specified set of parameters, and the model is then fit to this set of synthetic choice data, are the same parameters recovered? Related to parameter recovery: To what degree do model-based regressors depend on the model parameters - that is - how robust are the parametric regressors used for the EEG analysis robust to perturbations in the parameters themselves?

Response 17. We performed model- and parameter-recovery (the new Fig. 8 in the main text and the new Fig. S5 in Supplementary Material) and checked for the robustness of model-variables (model-based regressors; the new Fig. S6 in Supplementary Material).

More precisely, we generated data for 3 sets (with different random seeds) of 12 simulated participants using SurNoR with its parameters fitted to behavioral data (the same set of parameters as used for EEG analysis). The summary of the generated data is shown in the new Fig. 7 of the main text and the new Figures S3 and S4 of Supplementary Material.

We then fitted the parameters of SurNoR separately to each of the 3 sets of simulated participants - by applying the same optimization technique that we had used for fitting SurNoR parameters to behavioral data. The results are shown in the new Fig. S5 of Supplementary Material and show that the true parameters were successfully recovered with reasonable errors (compared to the curvature of the log-likelihood function). We also showed that the amount of error introduced in recovery of parameters does not change the model variables that are used for correlation with the EEG data (e.g., RPE and NPE) much - see the new Fig. S6 of Supplementary Material.

We used the same model-selection approach (the same pooling, number of folds, optimization method, etc.) and showed that SurNoR can be successfully recovered given the data of any of the 3 sets of the simulated participants (the new Fig. 8). The only difference between the model-recovery procedure and the model-selection procedure was that, for model-recovery, we included only the models that had turned out to be the best ones for the real data (Fig. 5) and allowed us to test the main three components (hybrid policy, novelty-seeking, and surprise modulation) of SurNoR: MF+S+N, Hyb+S+U, and Hyb+N. We made this choice because of the limited computational resources and for the sake of saving time - since for each random seed, the model-recovery took between 5 to 7 days, depending on the number of trials.

- The model offering the best fit to human behaviour is a hybrid MB/MF framework. But,

it's not clear to me where the MF and MB components have dissociable preferences. It might help clarify some of the model's complexity if the contributions made by some of the model's components were outlined.

Response 18. To address your point and to make the benefits of a hybrid policy more explicit, we analyzed two situations in the 1st episode of block 1 where the model-free and the model-based branches of SurNoR have different preferences over actions. The results are shown in the new Fig. S8 in Supplementary Material and are discussed in section "Qualitative differences between model-based (MB) and model-free (MF) branches of SurNoR" in Supplementary Material. There we show:

> The MB and MF branches of SurNoR have different preferences in different situations. Participants behave more MB in some situations and more MF in the others, and a hybrid policy can capture their behavior in both situations.

See the section and the corresponding figure for full explanation. In the main text, we point to this section when we discuss the relative importance of the MF and MB branches of SurNoR:

> Since SurNoR combines in its hybrid action selection policy a model-free with a model-based component (Fig. 6A2), we first wanted to analyze the relative importance of each of the two components in explaining the action choices of participants; see Supplementary Material for a qualitative comparison of these two components.

- It's not clear to me why a model-free Q-value scaling parameter is required? Are both systems not learning about the same environmental signals?

Response 19. Both systems learn the same environmental variables, and they must eventually converge to the same set of Q-values; however, the dynamics of learning are different. Let us consider the novelty Q-values. At the beginning, the novelty of all states are the same and equal to $\log |\mathcal{S}| = 2.40$. MF novelty Q-values are initialized to Q_{N0} which turned out to be very small (0.5 ± 0.35) according to the fit. MB novelty Q-values, however, are initialized to $\frac{\log |\mathcal{S}|}{1-\lambda_N}$ (i.e., the *a priori* solution to Bellman equations) which turned out to be very big (8 ± 1). Therefore, during the learning, the scaling parameter can play a significant role - which turned out to be the case with the fitted value of $5.8 \pm 0.2 > 1$.

- In addition to manually controlling novelty driven exploration, the model also includes manual manipulation of the MB/MF mixture parameter. What is this variation in mixtures capturing in the data?

Response 20. The main reason that we considered the MB/MF mixture parameter to be a function of time (i.e., piece-wise constant in this case) was because of the earlier evidence for such a phenomenon (Glascher, et al. 2010). We added a relevant paragraph in the Discussion section:

> Our results extend findings that humans use hybrid policies in two-stage decision tasks [5, 6] to the case of deep sequential decision tasks in the presence of abrupt changes. In general, the balance between model-free and model-based behaviors depends on multiple aspects and features of the task that humans are dealing with [3, 5, 70, 71]. For example, we observe that the model-based branch becomes more important when participants explore the environment to find the goal state, although

> the participants' behavior in our environment is always dominated by model-free action choices (Fig. 6A4). Moreover, the dominance of model-free behavior in such deep tasks does not exclude that humans used model-based planning in shallow tasks that are easily comprehensible thanks to a spatial arrangement of states or explicit instructions [72].

- Novelty seeking weights are re-applied at the start of block2. However, participants are unaware of the blocking structure - they only experience a surprise when they encounter the unexpected state. Why is novelty seeking re-invigorated for the start of block 2?

Response 21. We clarified our reasoning behind this choice by adding this paragraph to Supplementary Material:

> Surprise increases rapidly at the first mismatch that participants face in the 1st episode of the 2nd block, when they encounter an unexpected transition. We hypothesize that the unexpected transitions make them realize that something has changed in the environment and they do not know anymore a path to the goal state and need to re-explore the environment and search for the goal in the absence of any external reward; hence, we assume that the huge surprise signal triggers renewed exploration and we therefore set $\beta_N = \beta_{N2}$ for the 1st episode of the 2nd block.

Please note that we considered β_{N2} to be a free parameter, and possibly 0 or equal to β_{N1}.

- The transformation of Reward and RPE as applied to the EEG analysis deserves some consideration. What does it mean to detect signals correlated with R+RPE and R-RPE when RPE is defined in terms of reward and value expectation? By Reward do the authors mean experienced reward or reward expectation? Are there sufficient trials to perform a robust analysis of Reward with only 12 exposures to the rewarding stimulus?

Response 22. First, by Reward in the EEG analysis we mean the experienced reward. We clarified this in the text:

> Note that by 'Reward' variable we mean the goal-state indicator, i.e., it is equal to one when a participant visits the goal state and zero otherwise; importantly, it should not be confused with MB or MF reward values.

Second, since the Reward indicator and RPE are highly correlated (0.75), it is not possible to dissociate their contributions from each other - see Response 24. The variable R_+ has a correlation of 0.93 with both RPE and Reward (see Fig. S1 in Supplementary Material), and hence we can consider it as a variable relevant for the 'external' reward. From this perspective, the variable R_- might capture only redundant fluctuations, i.e., represents a sort of noise, and that might be why it does not contribute to the encoding model (see Fig. 10). In any case, the focus of our EEG analysis is not on the 'external' reward, and the reason we added reward-relevant variables was to make sure that the contributions of Surprise, Novelty, and NPE to the encoding model is not because of their correlation with reward-relevant variables. Please note that we do not make any strong conclusion about the contribution of R_+ to the encoding model, and that is why, in Discussion, we always use the term 'Reward/RPE' instead of Reward or RPE alone.

- The authors run many statistical tests, one for each timepoint in the EEG. I might be misinterpreting how this was done, but an FDR=0.1 implies that you expect 10% of your tests to be false positives. Does this leave a multiple comparisons issue with models run for each timepoint given the results reported? With 65 measurements, this implies around 7 false positive tests. It's hard to tell from the reported data, but it would seem there are roughly that number of positive tests for Surpris, R and RPE.

Response 23. We guess there has been a confusion between False Discovery Rate (FDR) and False Positive Rate (FPR). What we have used is False Discovery Rate (FDR), and FDR$\leq$ 0.1 implies that, on average, at most 10% of the *rejected* (i.e., discovered) hypotheses were null. Therefore, the number 65 is not really relevant in this case. Rather, the number of the rejected hypotheses matters, e.g., if we have rejected 10 hypotheses, then, we expect to have, on average, at most 1 false positive. Moreover, we have used the Benjamini and Hochberg algorithm to control FDR; the algorithm makes the assumption of independence between hypotheses (i.e., the worst case scenario in multiple comparison). Because most of our tests are on different points of time-series, our approach for controlling FDR is quite conservative.

- The authors orthogonalize novelty, surprise and novelty prediction error to R_+ and R_-. It would be preferable to allow variables to compete for variance as opposed to explicit orthogonalization.

Response 24. We repeated the regression analysis with the raw model-variables: we considered normalized Surprise, Novelty, NPE, Reward, and RPE as regressors. The results are reported in the new Fig. S2 in Supplementary Material with this explanation:

> The regression analysis without PCA and without orthogonalization (Fig. S2) yields results quite similar to the one with PCA and with orthogonalization (Fig. 10 in the main text); the main difference is that the significant positive correlation of a reward-related variable (R+) with the EEG amplitude in the 3rd time-window disappears. The reason is the very high correlation between Reward and RPE (Fig. S1A), which leads to imprecise estimation of regression coefficients (compare Fig. S2B and Fig. S2C with Fig. 10B and Fig. 10C in the main text). Please note that since the regressors in Fig. S2 are a linear combination of the regressors in Fig. 10, the adjusted R-squared is the same for both analyses (compare Fig. S2A with Fig. 10A in the main text).

Therefore, our main conclusion remains the same.
